# Unraveling the epidemiology of *Mycobacterium bovis* using whole-genome sequencing combined with environmental and demographic data

**DOI:** 10.3389/fvets.2023.1086001

**Published:** 2023-05-17

**Authors:** Gianluigi Rossi, Barbara Bo-Ju Shih, Nkongho Franklyn Egbe, Paolo Motta, Florian Duchatel, Robert Francis Kelly, Lucy Ndip, Melissa Sander, Vincent Ngwang Tanya, Samantha J. Lycett, Barend Mark Bronsvoort, Adrian Muwonge

**Affiliations:** ^1^The Roslin Institute, R(D)SVS, University of Edinburgh – Easter Bush Campus, Midlothian, United Kingdom; ^2^Centre of Expertise on Animal Diseases Outbreaks, EPIC, Edinburgh, United Kingdom; ^3^School of Life Sciences, University of Lincoln, Brayford Pool, Lincoln, United Kingdom; ^4^The Food and Agriculture Organization of the United Nations, Regional Office for Asia and the Pacific, Bangkok, Thailand; ^5^Royal (Dick) School of Veterinary Studies and the Roslin Institute, University of Edinburgh, Easter Bush, Midlothian, United Kingdom; ^6^Laboratory for Emerging Infectious Diseases, University of Buea, Buea, Cameroon; ^7^Department of Biomedical Sciences, Faculty of Health Sciences, University of Buea, Buea, Cameroon; ^8^Tuberculosis Reference Laboratory, Bamenda, Cameroon; ^9^Cameroon Academy of Sciences, Yaoundé, Cameroon

**Keywords:** *Mycobacterium bovis*, whole genome sequencing (WGS), genomic surveillance, zoonotic tuberculosis, phylodynamics, phylogeography, multi-host system, one health

## Abstract

When studying the dynamics of a pathogen in a host population, one crucial question is whether it transitioned from an epidemic (i.e., the pathogen population and the number of infected hosts are increasing) to an endemic stable state (i.e., the pathogen population reached an equilibrium). For slow-growing and slow-evolving clonal pathogens such as *Mycobacterium bovis*, the causative agent of bovine (or animal) and zoonotic tuberculosis, it can be challenging to discriminate between these two states. This is a result of the combination of suboptimal detection tests so that the actual extent of the pathogen prevalence is often unknown, as well as of the low genetic diversity, which can hide the temporal signal provided by the accumulation of mutations in the bacterial DNA. In recent years, the increased availability, efficiency, and reliability of genomic reading techniques, such as whole-genome sequencing (WGS), have significantly increased the amount of information we can use to study infectious diseases, and therefore, it has improved the precision of epidemiological inferences for pathogens such as *M. bovis*. In this study, we use WGS to gain insights into the epidemiology of *M. bovis* in Cameroon, a developing country where the pathogen has been reported for decades. A total of 91 high-quality sequences were obtained from tissue samples collected in four abattoirs, 64 of which were with complete metadata. We combined these with environmental, demographic, ecological, and cattle movement data to generate inferences using phylodynamic models. Our findings suggest *M. bovis* in Cameroon is slowly expanding its epidemiological range over time; therefore, endemic stability is unlikely. This suggests that animal movement plays an important role in transmission. The simultaneous prevalence of *M. bovis* in co-located cattle and humans highlights the risk of such transmission being zoonotic. Therefore, using genomic tools as part of surveillance would vastly improve our understanding of disease ecology and control strategies.

## 1. Introduction

In the last two decades, the increased availability, efficiency, and reliability of genomic reading techniques, such as whole-genome sequencing (WGS) techniques, have ignited a profound transformation in understanding disease ecology and epidemiology. This, coupled with improved statistical methodologies and high-performance computing, has enhanced our understanding of pathogen dynamics and evolution (1).

Techniques such as WGS can identify polymorphisms in the genetic material, which is generated by transcription errors that can occur to the pathogen while replicating within their host (2). As the pathogen is transmitted through the host population, the accumulation of polymorphisms in its DNA/RNA can be used as a “transmission signature”. Therefore, by tracking these mutations across bacterial genomes sampled in a host population, we are now able to infer transmission events between individual hosts, sub-populations, geographical areas, or species, while at the same time, we are able to gather insights about the evolutionary trajectory of a pathogen (2). Furthermore, when accurate spatial information on the sampled isolates is available, we can combine it with pathogen genetic data to disentangle the spatiotemporal dynamics of outbreaks, particularly in natural or other scarcely sampled animal populations (3).

Despite these advances, many challenges still exist, including the reconciliation between the temporal signal of outbreaks with pathogen mutations (4). *Mycobacterium tuberculosis* Complex (MTBC) members are clonal species, and therefore, recombination has been considered rare [although a recent publication showed otherwise (5)]. A few mutations are expected to occur for these species per year, generating little diversity during outbreaks in host populations. Consequently, there is inherent uncertainty in establishing infection patterns within the infected population and their associated infections. Therefore, combining genomic information with metadata is essential for accurate transmission chain estimation (4).

*Mycobacterium bovis*, a member of the MTBC group, is the etiological agent of animal or bovine tuberculosis (bTB) in bovids and other mammalians and of zoonotic tuberculosis (TB) in humans (6). Its infections are characterized by chronic disease, with or without a latent period, where infected cattle are hard to identify, making it hard to quantify potential infectious contacts (7). The estimation of *M. bovis* prevalence is often affected by several factors, including the inaccuracy of diagnostic tests (8), and the potential co-infection with other pathogens (9). Such challenges explain why *M. bovis* has only been successfully eliminated or controlled in a few countries. However, it still represents a significant threat to cattle industries and human health in many other countries. For example, zoonotic tuberculosis due to *M. bovis* is a major public health problem in low- and medium-income countries (LMICs), where close interaction between people and livestock is common and the access to pasteurized milk is limited (6, 10). Indeed, the magnitude of this burden is likely underestimated since human–animal transmission is predominantly via ingestion of infected products and it presents with a range of non-specific symptoms (11).

In Cameroon, *M. bovis* is circulating in the cattle population, both in the southern areas (12) and, in particular, in the northern regions, where a previous study on cattle sampled at four regional abattoirs showed a sampled population prevalence ranging from 2.75% (31 positive over 1,129 cattle inspected, Northwest) to 21.25% [34 over 160, North (13)]. Abattoirs surveillance, where carcasses are inspected for TB-like lesions, is the only surveillance strategy regularly implemented in the country; in Bamenda (Northwest region), Awah-Ndukum et al. (14) showed that the TB-like lesion in cattle increased in the period from 1994 to 2010.

Commonly to many LMICs, bTB control in Cameroon is also made difficult by the absence of detailed records on cattle population, by local rearing practices such as pastoralism which expose animals to contact with other herds and potential reservoir wildlife species, and by the transhumance cattle movements westward toward Nigeria, where the demand of meat is driven by a fast human population increase (15).

In a previous study, Egbe et al. (15) employed two molecular typing techniques to understand the relatedness of *M. bovis* strains circulating in the region. These are spoligotyping and MIRU-VNTR typing as follows: the former is based on the presence of multiple spacer oligonucleotides in the genome direct repeat region, while the latter is based on 12 loci containing variable numbers of tandem repeats of mycobacterial interspersed repetitive units (16, 17). Compared with WGS, these techniques consider a limited genome region and can be more subject to homoplasy (18). The results reported by Egbe (15) showed that most of the isolates belonged to the Af1 clonal complex (*n* = 250/total *n* = 255), while the remaining ones had an unidentified clonal complex. They also highlighted an unexpectedly high genetic diversity, as showed by the 37 sampled spoligotypes, of which, 19 were newly observed, and a total of 97 genotypes were obtained by combining spoligotypes with MIRU-VNTR (15).

While those techniques are instrumental to investigating potential infection clusters at a broader level, they can be limited for a more in-depth understanding of the spatiotemporal dynamics of the disease. This study aimed to fill these gaps and enhance our understanding of the *M. bovis* epidemiology and spatial dynamics in Cameroon using WGS. We applied novel phylogenetic techniques to determine whether there was endemic stability across Cameroon's cattle-rearing regions while examining the role of environmental and ecological variables and animal movements in the pathogen spread.

We used 91 high-quality *M. bovis* sequences obtained from cattle tissues sampled at regional abattoirs as described by Egbe et al. (13). After determining the single nucleotide polymorphisms (SNPs), we built a tree by joining the Cameroonian WGSs with other African sequences obtained from publicly available repositories, in order to understand how the sampled population fit in the continent context. Then, we ran a continuous space phylogeographical analysis with *BEAST* (19) on the Cameroonian sequences while testing different random walk diffusion models (20). This was possible because the origin village of the cattle tested at the abattoir was known for 64 *M. bovis* cattle isolates, allowing us to associate spatial coordinates with these sequences. Furthermore, we tested the association between the spatial pathogen distribution obtained with the georeferenced phylogenetic tree and environmental, anthropic, and ecological factors (21), and we finally ran a machine learning analysis to test whether the empirical cattle movement network (22) or other variables could explain the genetic diversity across isolates.

Our findings strengthen the call for improved *M. bovis* molecular surveillance in underrepresented regions and countries, to gather insights into potential patterns that can be missed when limiting the studies to areas of low genetic diversity, the consequence of strict control practices such as test-and-cull.

## 2. Materials and methods

### 2.1. Data collection

Four regional abattoirs were sampled between 2012 and 2013, in the Northwest (Bamenda), Adamawa (Ngaoundere), North (Garoua), and Extreme North (Maroua) regions of Cameroon (Supplementary Figure 1). As part of the regular operations, cattle carcasses were inspected for the presence of TB-like lesions. The tissues, including lymph nodes, of all animals with lesions and of some randomly chosen ones without lesions were collected to be cultured, and information about the animal (age, breed, and village of provenance, among others) was taken. A detailed description of the data collection and bacterial isolation can be found in the study by Egbe et al. (13). The DNA extraction was conducted in BSL 3 facilities (Tuberculosis Reference Laboratories in Bamenda, Cameroon), and the procedure is fully described in the study by Egbe et al. (15). Sequencing was also attempted for *M. bovis* isolates sampled in human hosts at the Bamenda hospital (Northwest region) during a cross-sectional study within the wider project. We reported a summary of the number of sampled animals and the number of *M. bovis*-positive ones in Supplementary Table 1.

### 2.2. Whole-genome sequencing processing

The sequencing was carried out at Edinburgh Genomic Facilities (University of Edinburgh). Samples were prepared with 1 TruSeq Nano 550 bp insert, 76 Pippin selected library from the supplied genomic DNA, while MiSeq v2 (Illumina) was used to generate 250 base paired-end sequences from the library to yield at least 11M + 11M reads (1 run) at 30x coverage. The output was read from a 4-lane Miseq. A total of 124 *M. bovis* WGSs were obtained (two from human hosts), while for nine isolates (one from human hosts), the sequencing failed.

We used an adapted *BovTB*-nf pipeline (23) for quality control. Reads were deduplicated using *fastuniq*, trimmed using *Trimmomatics* (24) (-phred33 ILLUMINACLIP:$adapters:2:30:10 SLIDINGWINDOW:10:20 MINLEN:36), and mapped to the reference genome using *bwa-mem2* (25). The mapped reads were filtered (-ShuF 2308 -) and sorted using *Samtools* (26), and then classified using *Kraken2* (27) (–quick) against a prebuilt Kraken 2 database [*Minikraken* v2 (27)]. The Kraken2 output was summarized with Bracken (28) (-r 150 -l S), and the top 20 species from the Bracken output were used to determine if the sample was contaminated with other microorganisms. Variants were called using *bcftools* (29) (–IndelGap 5 -e ‘DP < 5 && AF < 0.8'), and strain-specific SNPs were used for classifying whether the samples were *M. bovis* or not [custom script and differentiating SNPs taken from the study mentioned in MMMO (23)]. The percentage of coverage (>60%) on the reference, read depth (>10), and number of reads (> 600,000) were used to identify and remove samples with insufficient data. To curate aligned core-variants for the downstream phylogenetic analysis, variants were called and filtered using *Snippy* v4.6.0 (30), using the default settings (minimum coverage = 10, minimum VCF variant call quality = 100), with the *M. bovis* AF2122/97 genome (GenBank: LT708304.1) as the reference genome. Variants from repeated regions were removed [mask for repetitive regions taken from the study mentioned in MMMO (23)]. Core-SNPs were determined by the snippy-core function within *Snippy*, where a genomic position was considered to be a core-site when present in all samples. We defined “high-quality” sequences as the ones with genome coverage > 90% and reading depth > 10 (31), and we renamed the sequences with a string composed of the following information: host species, location (administrative subdivision or country, see Section 2.3), sequential number, and date.

For each sequence, the spoligotype and the clonal complex were retrieved from the study by Egbe et al. (15). In one case, a sequence was missing the spoligotype number; however, it was assessed with the *vSNP* pipeline (32). For all bioinformatics tools, we used the default settings unless stated otherwise.

We checked if divergent sequences belonged to other mycobacteria species. We tested the presence of regions of difference (RDs) 1, 4, 9, and 12 patterns (33) in the outlier samples, and raw reads from each sample were aligned to *M. tuberculosis* (NC_000962.3) with *Burrows-Wheeler Aligner* v0.7.17 (34) and sorted and indexed with *SAMtools* v1.10 (35). Primer flanking regions for the RDs on *M. tuberculosis* were determined by querying the sequences using NCBI web nucleotide BLAST with the default parameters (36), while the presence of RDs was manually determined by examining the read alignment in *Integrative Genomics Viewer* v2.14.1 (37).

### 2.3. Cameroonian *M. bovis* sequences in the African context

We obtained other *M. bovis* genomes from online repositories as follows: first, from the *Patric* (now, BV-BRC) dataset (38), and second, selecting the appropriate genomes among the ones listed by Loiseau et al. (39) and obtained from the EBI dataset (for details and references, see Supplementary Table 2). We selected all the available sequences sampled in Africa, in order to qualitatively detect potential genetic similarities between the sampled Cameroonian *M. bovis* population and other isolates from the African continent, and thus provide a broader context to our analysis.

When analyzing sequences from *Patric*, genomes were shredded into pseudo by *Snippy*, followed by the process of alignment and SNP identification described above. The core-SNP alignments were made with and without the other African genomes. We used the *iqtree* web server (40, 41) to compute a phylogenetic tree (*n* = 212), which included all the Cameroonian high-quality sequences (*n* = 91) and the other African ones plus the 1997 reference from the UK (*n* = 121).

### 2.4. Cameroonian sequences phylogenetic analysis

The quantitative analyses were performed on a subsample of the Cameroonian sequences, obtained after removing the non-cattle ones, the ones missing the geographical coordinates and potential outliers, i.e., isolates not clustering within the main Cameroonian clade. We initially joined the remaining sequences (*n* = 64) of the tree using the TN93 genetic distance model and the neighbor-joining (NJ) algorithm *ape* package (42) in *R* v4.0.5 (43),

with the sole purpose of estimating a temporal signal within the sample in *TempEst* v1.5.3 (44). We, then, used the sequences SNP alignment completed with sampling dates, to infer time-scaled phylogenetic trees using *BEAST* v1.10.4 (19) with the *BEAGLE* library (45) and evaluated the results with *Tracer* v1.7.2 (46). Since the sequences had associated geographical location metadata, we included latitudes and longitudes as an additional continuous space variable for phylogeographic inference.

To select the best model, we ran a series of exploratory models using an HKY (47) substitution model, similar to other studies (48–50) and a strict molecular clock. First, we sequentially selected the best continuous trait model and then the best bacterial population size model (tree prior). We tested the Brownian random walk, Cauchy Relaxed Random Walk (RRW), lognormal RRW, and Gamma RRW for the former, and constant population, exponential growth, and Bayesian *Skygrid* (51, 52) for the latter. In the exploratory *BEAST* runs, we chose a truncated (between 0 and 0.1) normally distributed clock rate prior, with mean and standard deviation set as the slope in the root-to-tip obtained in *Tempest*; the chain length was set to 10^8^ and sampled every 10^4^ steps. The models were compared using marginal likelihood estimation (MLE), with path sampling (PS) and stepping-stone sampling (SS), if they reached a satisfactory effective sample size (>200). Once the model features were selected, we ran a final one setting the chain length to 10^9^ steps and sampled every 10^5^ steps. In this case, we used the clock rate posterior of the selected exploratory model as a prior for the final model. The maximum clade credibility (MCC) tree was extracted with *TreeAnnotator* v1.10.4 (part of the *BEAST* suite), and clades were visually defined within the MCC tree branches. The MCC tree was plotted against the sequence spoligotype and MIRU-VNTR typing to visually assess the correspondence between molecular typing and clades.

### 2.5. Spatial statistics and environmental factors analysis

From the final *BEAST* run, we extracted a set of 100 trees from the posterior distribution and further analyzed using *seraphim* v1.0 (21, 53), to obtain the spatial spread statistics: branch velocity and epidemic wavefront. The former was calculated for each branch dividing the geographical distance from the origin to the destination nodes by the time branch time duration. The epidemic wavefront shows the geographical range of the epidemic over time: at each time, it is calculated as the geographical distance between the positions of the tree's estimated root and the most distant node (spatial distance wavefront) or accounting for the distance of nodes closer to the root (patristic distance wavefront).

Additionally, *seraphim* allows to statistically test hypothesis on the effect of environmental layers on the epidemic dynamics; the effect can either be of “conductance”, when the layer favors the pathogen diffusion, or “resistance”, when the layer hampers it. We tested nine layers as follows: elevation, cattle population density, human population density, two describing the road infrastructure (number of intersections and total road length), and four land cover types (waterbodies, forest, grassland, and grazeland, and other vegetation types, such as mosaic, shrub, and sparse vegetation). The original raster layers were downloaded from online repositories (see Supplementary Table 3 for the sources) and adapted to a 5 km × 5 km grid using *QGIS* v3.26.1. For each cell, elevation, cattle, and human populations were averaged for the 5 × 5 km grids, while road intersections were counted, and road lengths were measured starting from the same road original raster. For the land cover, each value represents the percentage of that cell covered by each land cover type. The original land cover raster included 38 different cover types. To ease computation, we selected the most relevant for the study and merged them into four layers as follows: waterbodies, forest, cropland/grassland, and other vegetation, including mosaic, shrub, and partial cover (Supplementary Table 4).

First, we ran a preliminary analysis on each variable, to determine if it could have played a role as conductance or resistance in the pathogen spread. For each of the 100 extracted trees, we estimated the correlation between dispersal duration and environmental distance. The results are summarized by two statistics as follows: the number of positive variable's coefficient of determination out of the 100 trees, and the number of positive *Q* statistic, calculated as $Q=\;R_{var}^{2}-R_{null}^{2}$, that is, the difference between the correlation R^2^ for the variable's raster and for a null raster, again calculated for each tree (53). For the analysis, we used two path models as follows: straight line (where the branch “weight” is calculated by summing the cell values through which the straight line passes) and least-cost path (where the branch “weight” is calculated by summing the values between adjacent cells along the least-cost path).

Once we identified the potential resistance or conductance factors, we performed 10 tree randomizations and calculated the statistics again. In this case, we used the Bayes Factor (*BF*_*e*_), calculated as *BF*_*e*_ = *p*_*e*_ */(1 – p*_*e*_*)*, where *p*_*e*_ is the probability that *Q*_*observed*_ > *Q*_*randomized*_. We used two criteria for tree randomizations as follows: (1) randomizations of node positions while maintaining the branches' lengths, the tree topology, and the location of the most ancestral node; and (2) randomizations of node positions while maintaining only the branches' lengths.

### 2.6. Genetic distance regression and role of the cattle movements

We, finally, tested which variables can better explain the genetic distances between the sampled *M. bovis* isolates, to understand the signatures of temporal, spatial, and demographic factors (54, 55). We ran this analysis using a Boosted Regression Tree (BRT) regression model (56) in *R* software [packages *dismo* (57) and *gbm* (58)], a very flexible tool that combines decision trees and boosting techniques (59). In this model, the dependent variable was the genetic distance between *M. bovis* strains, expressed as SNPs. We tested a total of 28 relational variables, calculated for each pair of isolates (Supplementary Table 5). Except for the temporal and spatial distance (which were calculated from the original isolate metadata) and a binary variable indicating whether two sequences have the same spoligotype, MIRU-VNTR, and clade (yes/no), the other variables are associated with the *M. bovis* isolates administrative subdivision.

We built two subdivision-level contact networks. The first one is a spatial network where nodes represent subdivisions, and edges between them are positive if they share a border. This network is undirected (edges are not directional) and unweighted (all edge values are set to 1). For this network, we computed six variables to be associated with each pair of isolates: degree and betweenness centrality (60) of both isolates' subdivisions; shortest path and a binary variable indicating whether the two subdivisions belonged to the same network's community.

The second network represented the cattle movements, and edges correspond to the number of animals moved between subdivisions over a year. We built this network by aggregating the empirical data collected by Motta et al. (22), which originally reported the monthly number of cattle exchanged between markets. For this network, we computed eight variables as follows: degree, strength, and between centrality of both isolates' subdivisions; shortest path and the same community binary variable. The degree counts the number of each subdivision's connections, while the strength is the sum of the number of cattle moved to and from each subdivision. All networks' metrics were computed using the *R* package *igraph* (61).

Once we computed all the variables (the full list is presented in Supplementary Table 5), we trained the BRT model using 75% of the observations, while the remaining 25% were used for testing. We evaluated the models based on pseudo-R^2^ and Root Mean Squared Error (RMSE) on the test dataset. These were both calculated using the package *caret* (62). For BRT, the relative influence of the variables is determined by the times each variable is selected to split the data in a decision tree, which, in turn, is weighted by the improvement in the model fit that resulted from the variable being used at each split (56). All models were fitted with a 10-fold cross-validation. The BRT algorithm has two main parameters as follows: the learning rate, which controls the contribution of each tree to the final model, and the tree complexity, which corresponds to the number of nodes in the tree. We ran some preliminary tests to tune the BRT in order to improve the predictions. Finally, we set the learning rate to 0.05 and the tree complexity to 8.

## 3. Results

### 3.1. Cameroonian sequences in the African context

**Figure 1:**
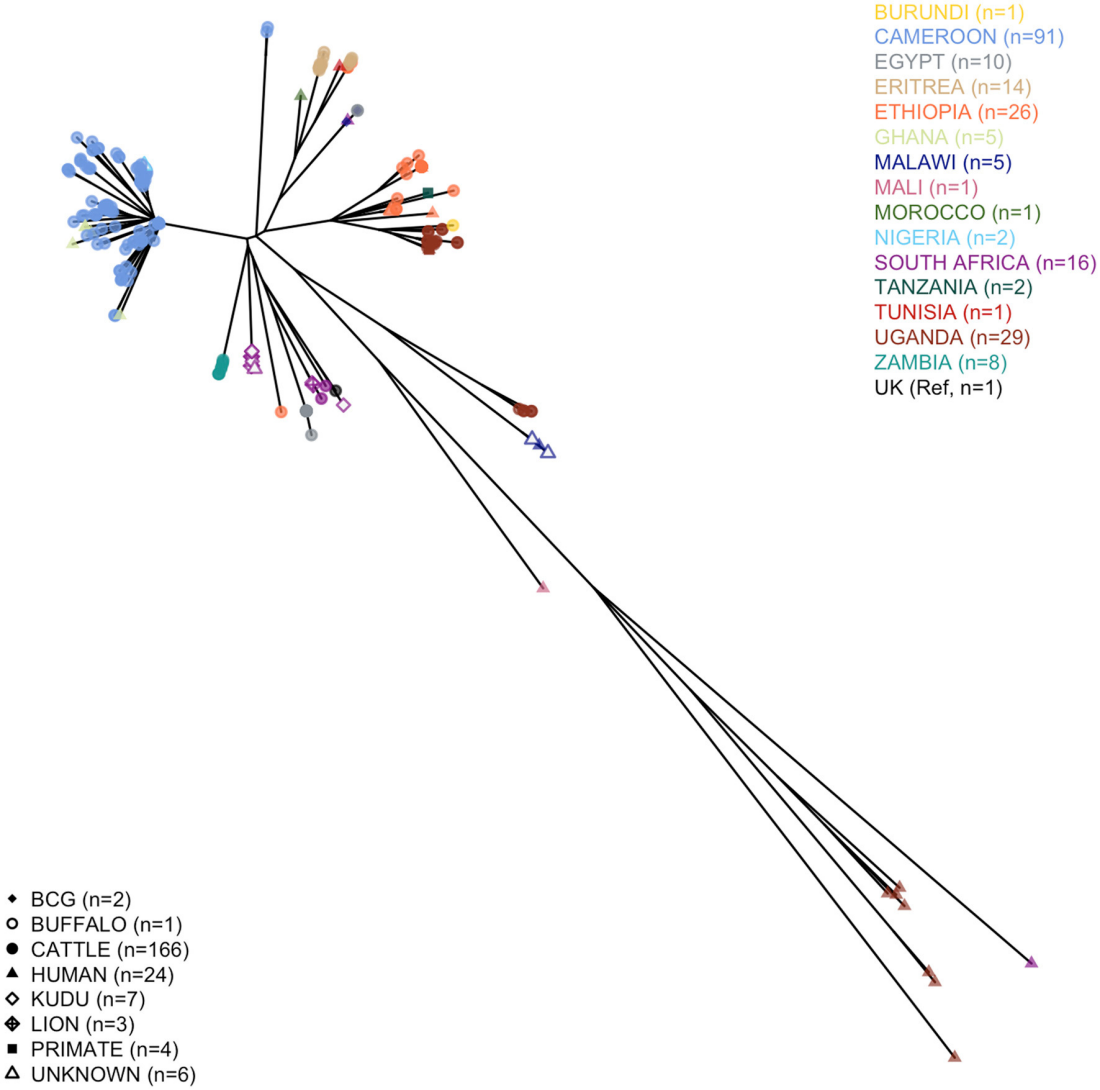
Phylogenetic tree of the African *Mycobacterium bovis* whole-genome sequences considered in the study. The tree includes 91 high-quality Cameroonian sequences, 101 from the EBI dataset, 20 from Patric, and the 1997 UK *M. bovis* reference.

We analyzed 124 *M. bovis* sequences (nine of the original 133 failed), with 91 having enough read depth and genome coverage to allow further analyses (see Supplementary Table 6 for further details). Two of these sequences came from isolates sampled humans, while for a third, the sequencing failed. One of the excluded sequences was marked as not *M. bovis*, and based on the presence of the four RDs 1, 4, 9, and 12 patterns (33), it was likely *M. tuberculosis*. All the high-quality *M. bovis* sequences were merged into a tree, with other 22 obtained from the *Patric* dataset, 99 from EBI, and the 1997 UK Reference to provide a continental context. The qualitative phylogenetic tree, in Figure 1, shows that most of the Cameroonian sequences (two of which obtained from human tissue samples) cluster with the Ghanaian human samples, and two Nigerians recovered from unreported hosts. All human samples from West Africa cluster with cattle sequences, except for the Malian human sequence. Most sequences (*n* = 89) belonged to Af1 clonal complex, and except for one, the spoligotypes were already known; for the other, we identified a new pattern (hex code: 6F-1F-5F-7F-BF-40). Being characterized by the absence of spacer 30, this spoligotype was considered Af1 (63). The dominant spoligotype was SB0944 (*n* = 32/89).

Two outlier sequences did not cluster with the rest of the sampled Cameroonian population. Their average distance from the rest of the Cameroonian population (respectively, 235 and 231 SNPs) was slightly higher than the average distance of the 1997 UK Reference from the Cameroonian isolates (222 SNPs), and they did not cluster with any other WGS sequence sampled in Africa (Figure 1). For both outlier sequences, the spoligotype was SB2332, found for the first time in Cameroon and submitted for classification at www.Mbovis.org by Egbe et al. (15). Following Warren et al. (33), we tested the presence of RDs 1, 4, 9, and 12 patterns, finding only the first one, confirming that they are likely *M. bovis*. We compared this spoligotype pattern with all the others from the *http://www.Mbovis.org* database, and we identified four patterns differing by two spacers as follows: SB0858 sampled in Spain (64) (different in spacers 20 and 22), SB1102 sampled in Chad (63) and Cameroon (12) (different in spacers 33 and 34), SB2333 reported by Egbe et al. (15) (different in spacers 22 and 34), and SB2691 sampled in France (not found in publications, different in spacers 20 and 34). We also identified 11 patterns differing by three spacers, sampled in France (65), Portugal (66), and Spain (64).

### 3.2. *M. bovis* evolutionary time scale in Cameroon

**Figure 2:**
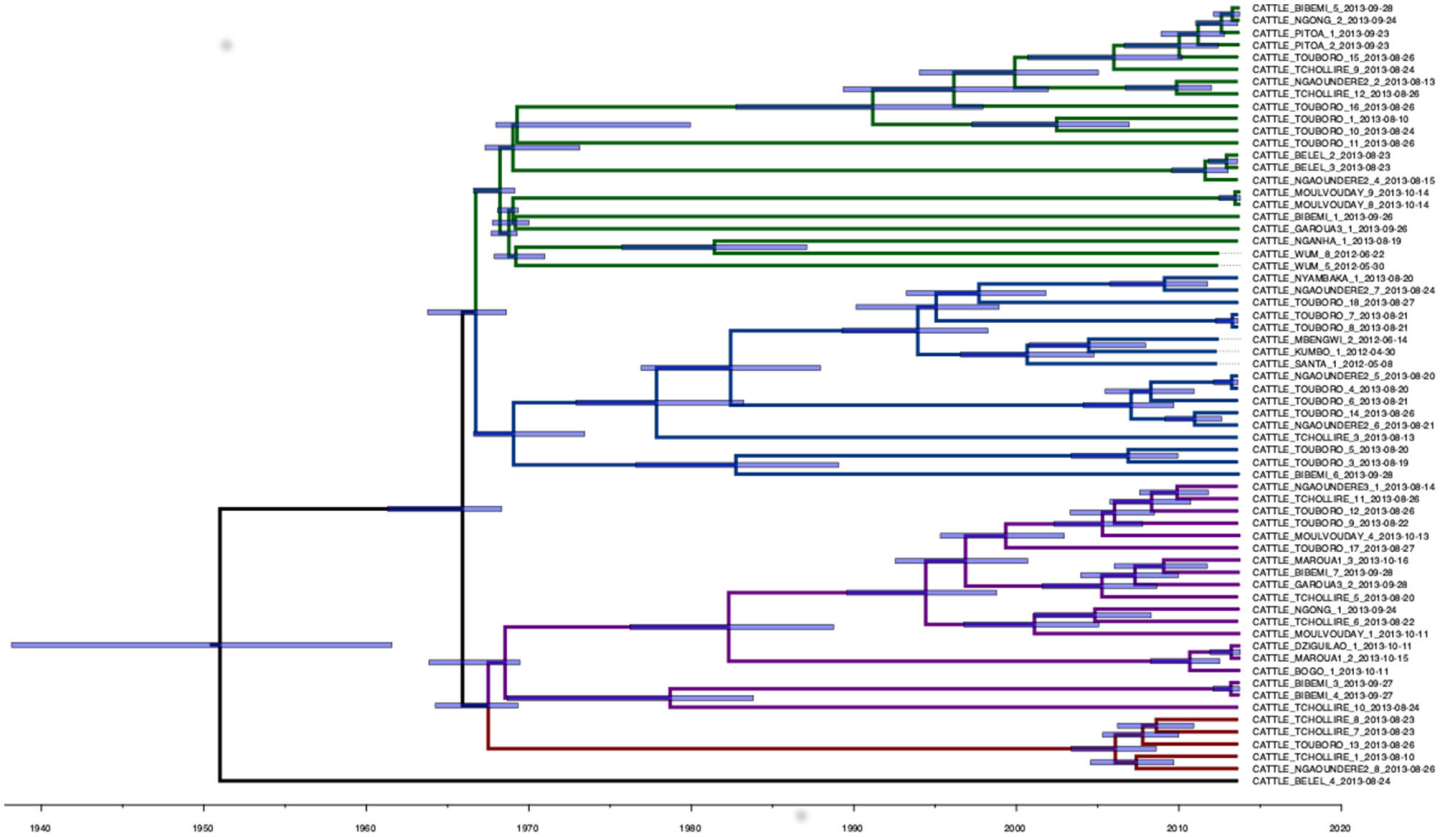
Phylogenetic time-scaled MCC tree of the 64 high-quality *M. bovis* whole-genome sequences sampled in Cameroon in 2012 and 2013. The blue transparent bars represent the 95^*th*^ HPD of the internal node dates, while the branch colors represent different clades: 1 (green), 2 (blue), 3 (purple), and 4 (red). A non-time scaled tree showing the genetic distance between the 64 sequences is presented in Supplementary Figure 6.

A total of 1,540 SNPs were determined from the *Snippy* core-SNP analysis on Cameroonian *M. bovis* genomes (Supplementary Figure 2). This was reduced to 1,106 SNPs when the dataset was reduced to 64 samples with complete metadata and excluding the non-cattle ones (two sampled in humans), which were used for the downstream quantitative analysis. The median SNP distance among the remaining high-quality sequences was 70 SNPs (mean 68, range from 0 to 144, 2.5^th^ and 97.5^th^ quantiles 14 and 118). For two cattle (one from Bibemi and the other from Touboro), two *M. bovis* isolates sequenced were available (obtained from different tissues). In both cases, the two strains were identical (Bibemi, 3 and 4; Touboro, 7 and 8, Figure 2), which suggests a single infection disseminated in different organs rather than two separate infections.

**Figure 3:**
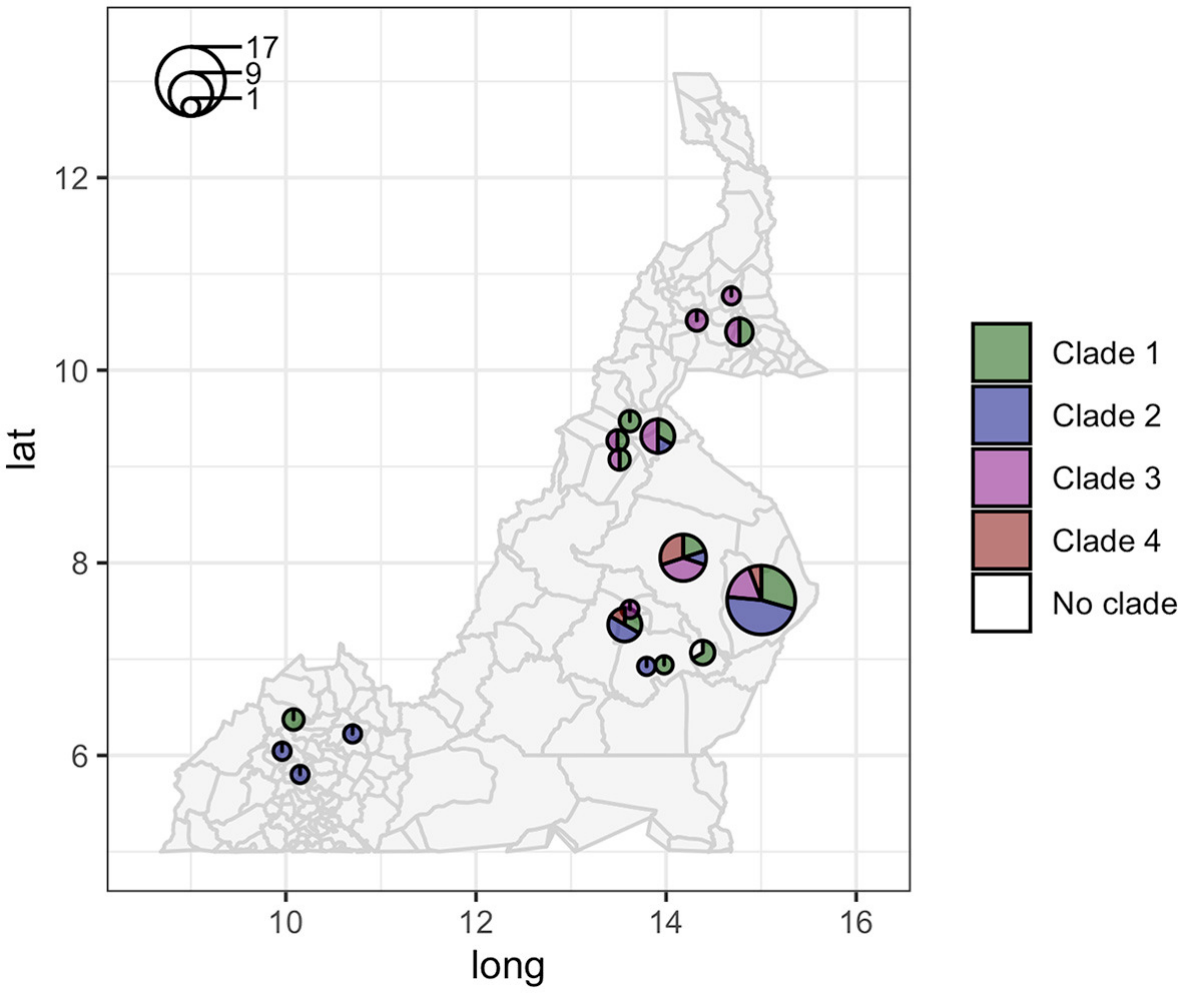
Geographic distribution of the 64 high-quality *M. bovis* whole-genome sequences in Cameroon. Circle sizes correspond to the number of sequences per administrative subdivision, and colors represent different clades (clade 1 green, clade 2 blue, clade 3 purple, and clade 4 red).

**Figure 4:**
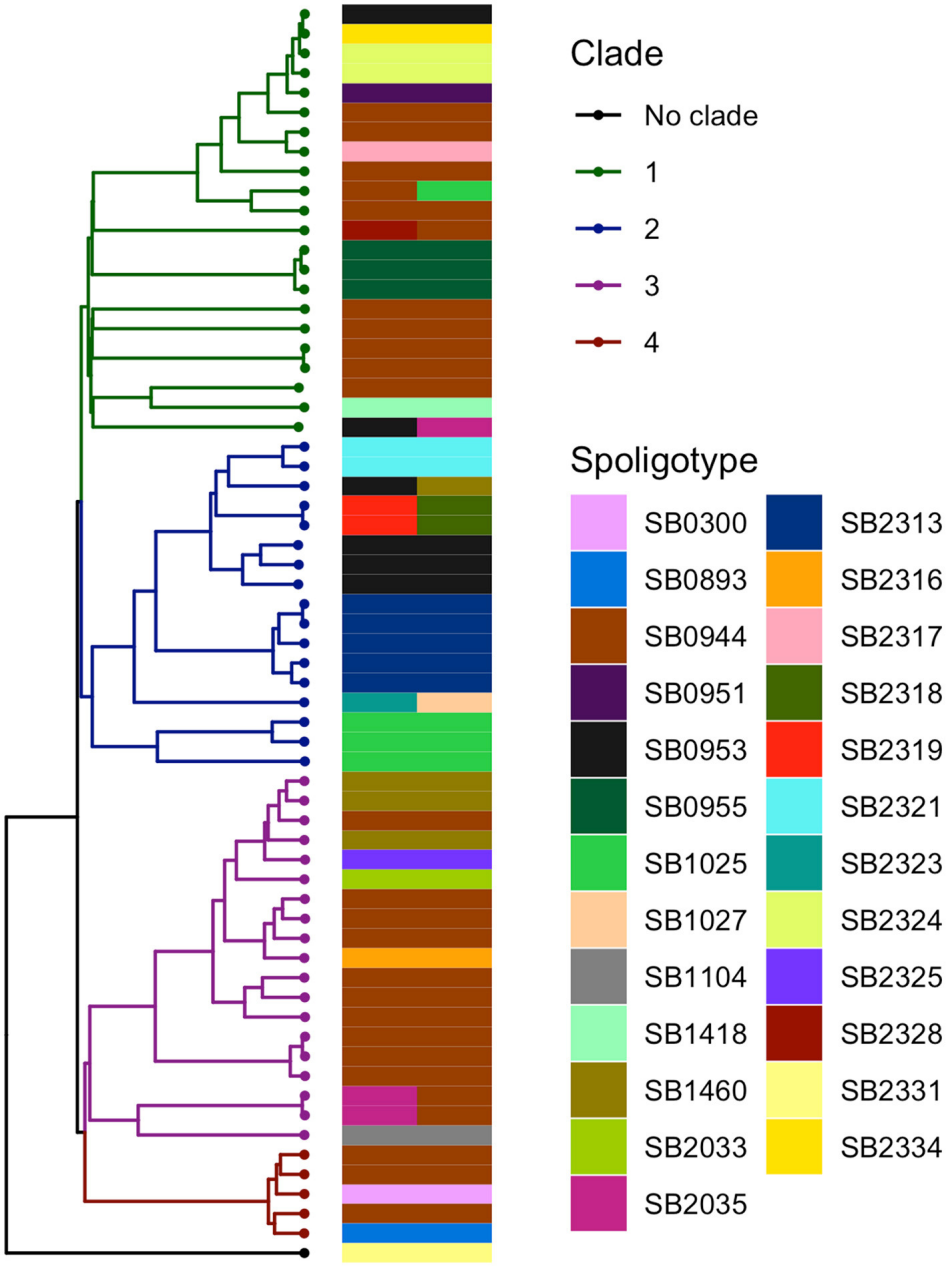
Visual comparison between the *M. bovis* phylogenetic time-scaled MCC tree and the spoligotypes obtained by Egbe et al. (15). A total of 10 sequences were associated with two spoligotypes because multiple samples from the same animal (up to three) were submitted for spoligotyping.

The analysis in *Tempest* showed a slightly positive temporal signal (coefficient of determination 0.11 and correlation coefficient 0.33) and a slope of 1.267 × 10^−2^ (Supplementary Figure 3). We used a sequential approach in *BEAST* to select the best spatial model and bacterial population models. Based on the MLE estimation of the exploratory models (Supplementary Table 7), we determined the best model included a Gamma Relaxed Random Walk (RRW) spatial model (first step of the sequential analysis) and the SkygGrid population model (second step). The final *BEAST* model was run with 10 bins and a cutoff of 400 years. The population trend is shown in Supplementary Figure 4. The model estimates suggest that the mean age of the root was in July 1950 (95^th^ high-posterior density, HPD, April 1938–August 1961), while the average clock rate was 1.32 × 10^−7^ substitution/site/year (95th HPD 1.20 × 10^−7^ – 1.44 × 10^−7^). The maximum clade credibility (MCC) tree is presented in Figure 2, which also shows the division in four clades as follows: clade 1 (green, 22 isolates), clade 2 (blue, 17 isolates), clade 3 (purple, 19 isolates), and clade 4 (red, five isolates). One sequence was excluded from all clades (Belel, 4; Figure 2, reported as “no clade” in the figures). The geographical distribution of the clades is presented in Figure 3, showing the number of *M. bovis* isolates per administrative subdivision, which ranged from 1 to 17 (see Supplementary Table 8 for the number of isolates per clade by regional abattoir). In Figure 4, we superimposed the MCC tree with spoligotypes; the most prevalent spoligotype, SB0944, occurred 26 times (out of 64 sequences) and was present in three of the four clades. The second most prevalent spoligotypes were SB0953 and SB2312, the first occurring five times in two clades and the latter occurring five times in one clade only (clade 2). We also superimposed the MIRU-VNTR types, as shown in Supplementary Figure 5. The most prevalent MIRU-VNTR type in the sampled population was V89, which occurred nine times; V82 and V37, respectively, occurred six and four times; and V81, V76, and V100 all occurred three times. Seven MIRU-VNTR types occurred two times, while 39 types occurred only once.

### 3.3. Spatiotemporal pathogen expansion

**Figure 5:**
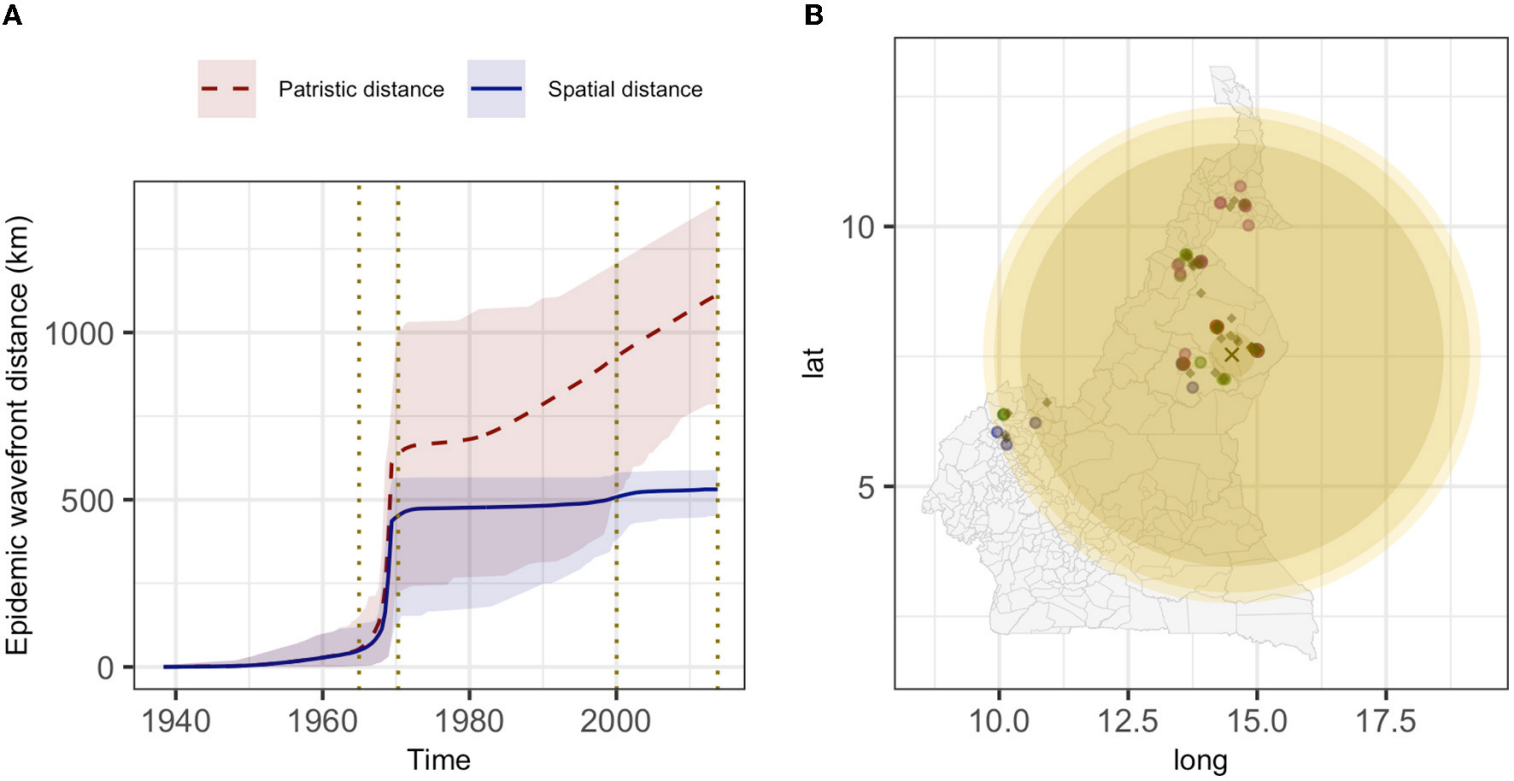
The estimated epidemic wavefront over time **(A)** and the expansion of the epidemic wavefront on the map **(B)**. **(A)** mean (lines) and 95^th^ HPD (shades) of the epidemic wavefront spatial distance (blue) and patristic distance (red) over time. **(B)** different yellow shades represent the epidemic wavefront at a sequential point in time [marked by vertical dotted lines in **(A)**], and lighter shades of yellow correspond to more recent expansion; the estimated tree's root location is indicated by the black cross, diamonds represent the internal nodes estimated locations and circles the sampled isolates (colored by clade: 1, green; 2, blue; 3, purple; and 4, red).

**Figure 6:**
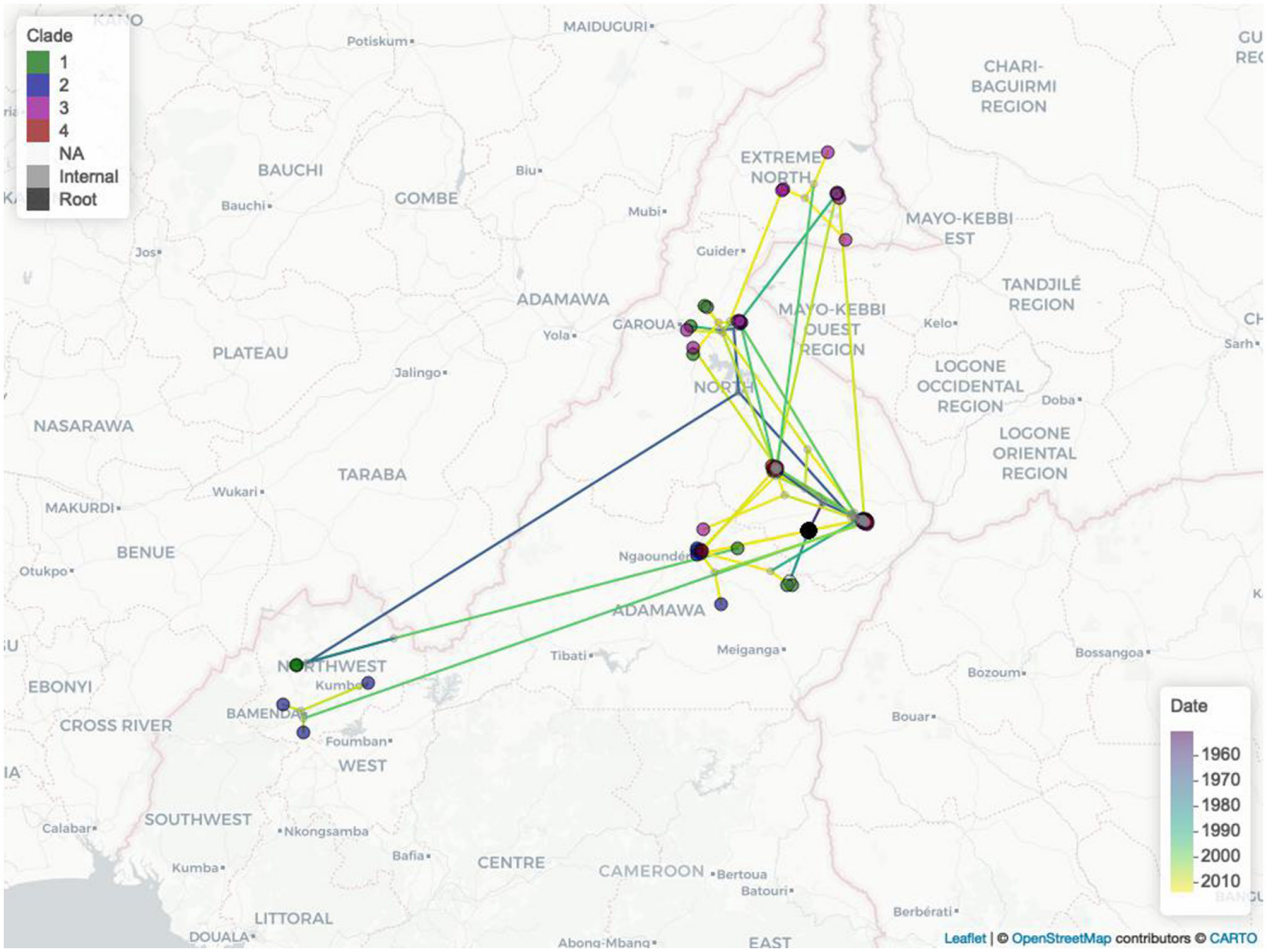
The Cameroonian *M. bovis* epidemic estimated expansions in space and time. Nodes are colored by clade (1, green; 2, blue; 3 purple; 4, red; no clade, light gray; internal nodes, dark gray; tree root, black), while the branches are colored by estimated movement date from 2007 (purple) to 2013 (yellow).

The estimated mean branch velocity was 53.1 km/year (95^th^ CI 18.4–219.0, temporal trend presented in Supplementary Figure 7). The wavefront statistics in Figure 5 suggest that the pathogen expansion was slow until the mid-1960s but accelerated thereafter to reach the entire study area, with a slow but constant expansion in the following period. This is reflected in an increase in the branch velocity at the same time (Supplementary Figure 7), which is approximately the period when the branches formed the observed clades (Figure 2). The timing of the different branches in space is presented in Figure 6 (95^th^ HPD in Supplementary Figure 8, with nodes colored by estimated/observed date).

We tested the association between nine geographical variables with the dispersal duration. Table 1 shows the results obtained using the straight line and the least-cost path models, and the latter run considering the variables as potential conductance or resistance factor. A total of six variables resulted in a significant association (positive coefficients for all at least 95 out of 100 trees and above 75% of positive *Q*) as follows: mosaic, shrub, and other vegetation covers (with both path models as resistance in the least-cost one); forest cover, elevation, and waterbodies cover (all as conductance); and cattle density (as resistance). However, when their statistical significance was tested through randomization, only forest cover and elevation (both as conductance) showed a Bayes factor significance [≥ 3 (67)]. The result was robust against two different tree randomization algorithms for the forest layer, while for the elevation, this was true only when maintaining the branches' length and excluding the other tree topological characteristics.

**Table 1:** The results of the analysis on nine spatial variables, assuming two path models, straight line and least cost, and for the least cost path, whether the variable worked as a conductance or resistance.

**Variable**	**Type**	**Path model**	**Number of positive coefficients**	**Number of positive Q statistic**	**Mean Bayes factor**	

					**(Randomization #1)**	**(Randomization #2)**
Mosaic_shrub_otherv	Resistance	Least cost	100	99	1.89	1.83
**Forest**	**Conductance**	**Least cost**	**100**	**96**	**3.66**	**3.66**
Mosaic_shrub_otherv	NA	Straight line	100	89	0.62	1.32
**Elevation**	**Conductance**	**Least cost**	**100**	**88**	**2.39**	**3.00**
Waterbodies	Conductance	Least cost	100	87	1.10	0.97
Cattle_density	Resistance	Least cost	99	77	2.49	2.88
Cattle_density	NA	Straight line	100	73	Not run	
Cattle_density	Conductance	Least cost	100	70	Not run	
Grassland_cropland	Resistance	Least cost	100	66	Not run	
Grassland_cropland	NA	Straight line	100	56	Not run	
Mosaic_shrub_otherv	Conductance	Least cost	100	44	Not run	
Roads_intersections	Conductance	Least cost	100	42	Not run	
Waterbodies	Resistance	Least cost	100	38	Not run	
Waterbodies	NA	Straight line	100	27	Not run	
Grassland_cropland	Conductance	Least cost	100	15	Not run	
Forest	Resistance	Least cost	100	12	Not run	
Elevation	NA	Straight line	100	7	Not run	
Forest	NA	Straight line	100	3	Not run	
Pop_density	Conductance	Least cost	99	40	Not run	
Elevation	Resistance	Least cost	99	15	Not run	
Roads_length	NA	Straight line	97	0	Not run	
Pop_density	NA	Straight line	96	16	Not run	
Pop_density	Resistance	Least cost	96	7	Not run	
Roads_length	Conductance	Least cost	92	11	Not run	
Roads_length	Resistance	Least cost	59	0	Not run	
Roads_intersections	NA	Straight line	56	1	Not run	
Roads_intersections	Resistance	Least cost	6	1	Not run	

The results show the number of positive coefficients for the 100 sampled trees, the number of positive Q statistics, and the mean Bayes factor calculated over 10 randomizations, testing two algorithms as follows: (1) randomizations of nodes positions while maintaining branches lengths, tree topology, and location of the most ancestral node; and (2) randomizations of nodes positions while maintaining only the branches lengths. The randomization algorithm was not run for spatial variables not significant in the preliminary analysis, variables significant in the randomization analysis were marked in bold.

### 3.4. Factors associated with genetic distance

**Figure 7:**
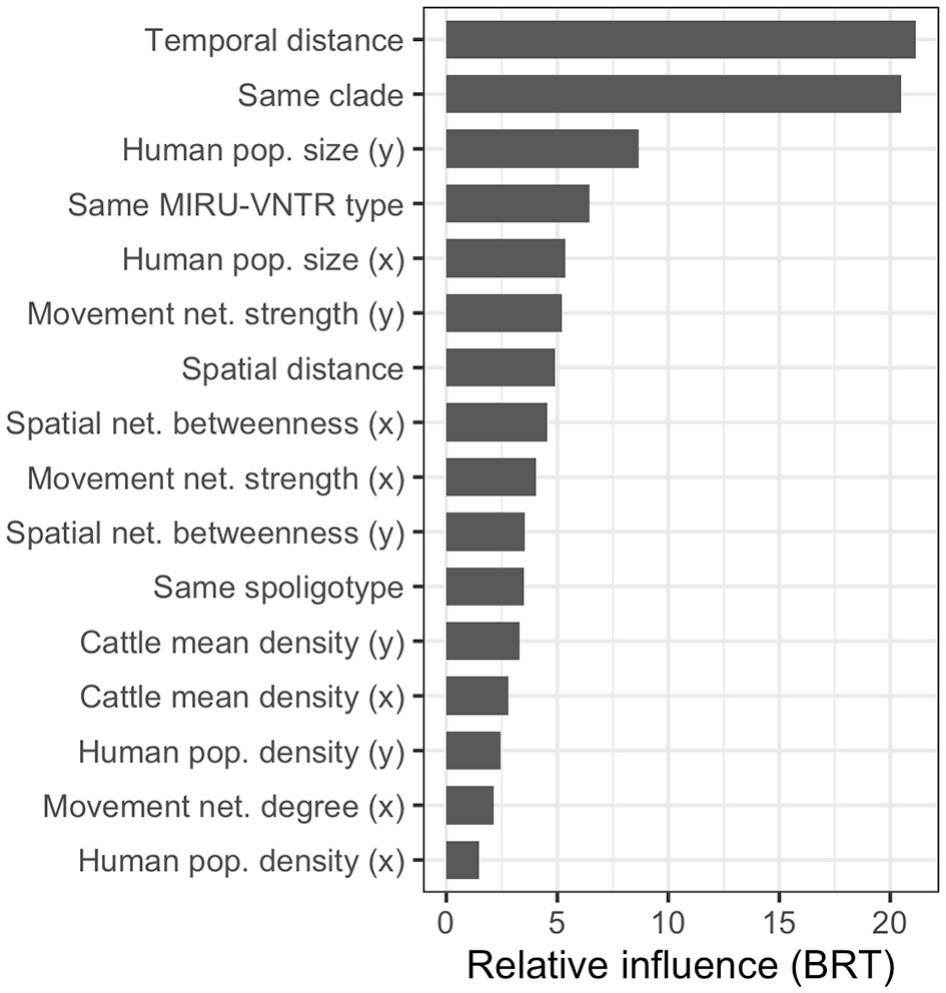
Relative influence of the most relevant variables in the simplified boosted regression tree (BRT) model used to explain the SNP distance between the 64 high-quality *M. bovis* isolates. Many variables are calculated between isolates pairs, *x* refers to the oldest isolate's subdivision, and *y* to the youngest one.

The RMSE of the boosted regression tree BRT model ran using all 28 variables was 20.23, while the R^2^ was 0.450. We simplified the model using the *dismo* package, which tests the performance of the model by dropping the less important variables with a procedure similar to backward selection in regression (56). The algorithm brought to eliminating 12 variables (see Supplementary Table 5); nonetheless, the model run using the remaining 16 variables performed very similarly to the original one (RMSE = 20.22 and R^2^ = 0.452). Therefore, we used the latter to calculate the variable importance (Figure 7).

As expected, the most relevant variables were the temporal distance between the samples (first), and the binary variable indicating whether the two *M. bovis* isolates belonged to the same clade in the MCC tree (second). The variables describing the subdivisions' population were also relevant in the model (population.y, third, and population.x, fifth), as well as whether the two isolates shared the same MIRU-VNTR (fourth). This was more relevant than the two isolates shared the same spoligotypes (11th), suggesting the former was more useful to discriminate closer *M. bovis* strains. The market movement network strength (i.e., the number of cattle moved from/to a subdivision) was the most important (sixth and ninth) among network-related variables, while the betweenness (eighth and 10th) was the only spatial network variable retained in the simplified model. Interestingly, when both variables were selected for the same metric, the one related to the youngest isolate (marked by *y*) was always preferred to the one related to the oldest isolate (marked by *x*). The partial dependency plots, showing the relationship between SNP distance and variables, are presented in Supplementary Figure 9.

## 4. Discussion

We sought to unravel the characteristics of the spread of a pathogen with zoonotic potential in time and space to improve our understanding and inform control and preparedness strategies. Our basic premise is that the accumulation of mutations in the pathogen's genome can be used as signatures of transmission events from host to host across time and space. Within space, the environment can create barriers that influence the population dynamics of diseases, i.e., altering host-to-host and pathogen–host interactions has direct effects on the genetic structure of the pathogen (68). The availability of high-throughput genomic techniques means we can interrogate the structural changes linked to the environment over time to gain critical insights into how the epidemic has evolved. In this study, we aimed to characterize *M. bovis* samples from cattle in Cameroon using genetic and demographic data to understand whether the pathogen is in a stable endemic state and the influence on the spread dynamic of environmental and ecological factors and cattle movements.

### 4.1. Evidence of dynamic endemicity

An important question was whether the *M. bovis* outbreak in North Cameroon was in a steady state, at an endemic equilibrium, or if it was expanding. Determining whether a pathogen is endemic has implications on risk perception and consequently on resource allocation. At the same time, the chances of zoonotic transmission are likely to be higher in the case of endemicity. In our analysis, the Bayesian model estimation with SkyGrid as a population model showed an increasing pathogen effective population size, corresponding to a constant increase in the disease velocity after the sudden jump during the mid-to-late 1960s. This suggests that the pathogen is not in a state of endemic stability, instead, it has been expanding at various rates over the years. This is in agreement with a previous publication using spoligotypes and MIRU-VNTR (15) and with the study by Awah-Ndukum et al. (14). The expansion of *M. bovis* might represent an issue for livestock and humans, particularly as we showed that the bacterium is circulating in both. At the moment, disease control in the area is absent, while, on the other hand, the dairy industry in Africa is generally expanding. A lack of widespread milk pasteurization could lead to an increase in zoonotic TB cases, which already represent a problematic issue in the region (11).

### 4.2. Genetic diversity of *M. bovis* in Cameroon

We observed a high diversity of *M. bovis*, confirming earlier observations with molecular typing techniques, providing less granular information (15), and considering the short time span of the sampling campaign and the small sample size. This contrasts with areas such as Great Britain and other European countries, where strict control measures, such as routine testing and stamping out of positive individuals, have been in place for decades. These measures could have resulted in a genetic bottleneck which hampered the pathogen's genetic variability, in particular by reducing the time a pathogen has to develop inside a domestic host and, therefore, the likelihood of substitutions in the DNA. As an example, Crispell et al. (55) reported a similar SNP distance range (0 to 150), albeit across a much bigger sample (*n* = 230), with a lower median (20 SNPs) and with isolates dating back two decades, while in a similar size monophyletic outbreak (*n* = 64), Rossi et al. reported a maximum SNP distance of only six SNPs (54). In Spain, Pozo et al. (69) found a similar SNP distance average and range (62 and 0 to 150) in a bigger *M. bovis* population, sampled in both cattle and wildlife over 13 years. It is noteworthy that high diversity can be associated with dynamic epidemiology and not with endemic stability.

All 64 core isolates belonged to the clonal complex Af1, which was observed in the region in previous studies (63). The most common spoligotype, SB0944, was found by Müller et al. (63) as the most prevalent in West Africa and considered the original of the Af1 clonal complex. Our findings also suggest zoonotic transmission in West Africa, as sequences recovered from humans in Cameroon and Ghana clustered with Cameroonian cattle *M. bovis* isolates (70). Because it is known that zoonotic TB represents a minoritarian but still crucial part of all TB cases in Africa, these results strengthen the case for One-Health approaches to control, which involve humans, livestock, wildlife, and environmental health (11, 71). Except for the one sequence in Mali and the two Cameroonian outliers, all the sequences from West Africa clustered together, hinting at high connectivity likely caused by cattle movements throughout the area, as previously shown by another study (72). Our results showed that the areas with the highest *M. bovis* diversity were in the Adamawa and North regions, both reporting all the clades identified by the maximum clade credibility (MCC) tree. All clades were also sampled in the towns of Touboro and Tchollire, both located in the North region but close to the Adamawa border. Previous studies reported that this area receive cattle from neighboring country as part of the transhumance migration, suggesting that cattle movements and markets play an important role in defining the dynamics of the pathogen, and therefore influencing its genetic diversity (15, 22). The Northwest region was underrepresented in the sample, with only five high-quality sequences on 31 infected cattle detected at the abattoir. This inherently reduces the level of diversity, which is far lower than reported using spoligotypes and MIRU-VNTR (15).

Despite covering a smaller portion of the genome and the higher occurrence of homoplasy with respect to WGS, in other contexts, spoligotypes have been used as a proxy cluster or to narrow down potential transmission within the study population (55, 73). Our results showed that this cannot be conducted for areas with high diversity such as the one we considered, as we observed little correspondence between the MCC tree branches and the spoligotypes. Similarly, other studies pointed out the limitations of such typing techniques (18), in the case of an expanding infection where transmission is steadily ongoing, compared with point-source ones (74). The high SNP distances among the sampled isolates also precluded the use of methods to infer direct transmission between hosts (7, 75).

When considering the entire sampled population, therefore including the sequences with incomplete metadata, we found two of the 91 sequences not belonging to the clonal complex Af1. In their spoligotype pattern (SB2332), we noted the absence of spacer 21 (76), and the closest relatives analyzed by Loiseau et al. (39) were identified as part of the clonal complex Eu2, including isolates sampled both in south-western Europe (SB0837, SB1090, and SB1308) and West Africa [SB1102, isolated in Cameroon as well (12)]. We can, then, speculate that these sequences likely belong to Eu2 as well, although we could not exclude one of the “unknown” clonal complexes identified by other studies (39, 77). Further development on this point was beyond the scope of this study, as we focused on the 64 core sequences to gather insights into the pathogen dynamics in the area.

### 4.3. Tracking the spread of *M. bovis* in Cameroon

We acknowledge that our estimates for the most recent common ancestor (MRCA) have a wide credible interval around it (23 years). This uncertainty is likely due to the short duration of the sample collection campaign, which also generated a weak temporal signal, although the coefficient of determination was similar to other *M. bovis* studies in highly sampled populations (54, 55). Nonetheless, our estimates coalesce around 1950, suggesting that the pathogen has been spreading in the area for at least six decades at the time of sampling. For the same reason, the estimated clock rate was higher than others in the literature but in the same order of magnitude (0.67–1.26 × 10^−7^, *n* = 2625 (39)].

The estimated MCC tree located at the most recent common ancestor (MRCA) in Touboro (North region), and from there, a rapid expansion of the outbreak reached most of the study area by the early 1970s. From the estimated origin, the pathogen likely spread first northward to Garoua (in the same region) and westward to the Northwest region, and later to the Extreme North and Adamawa regions and again to the Northwest.

The results of the spatial factor analysis showed that forest cover and elevation were the only significant ones, both acting as “conductance”. Forest cover could be a proxy for potential wildlife interactions, as *M. bovis* is known to be quite effective in spreading at the wildlife–livestock (and humans) interface (71, 78). The elevation as conductance was counter-intuitive; however, this could be linked to cattle movements in pastoralist communities within the plateau located in the study area. This is important because, if confirmed, altitude could be used as a proxy for the missing pastoralist movements.

Our regression model performed reasonably well, although the amount of variability explained was below 50%. However, our objective was to understand which variables could better explain the genetic distance between *M. bovis* isolates, expressed as SNP distance. Except for the isolates between temporal distance and clade, the demographic variables were the most effective in explaining SNP distance, particularly the administrative subdivision human population size. These variables had a negative effect on the SNP distance, meaning that a smaller population was associated with a close relatedness of the *M. bovis* strains. This could be an effect of the population distribution on the country because the northern regions, where cattle are most concentrated, are less populated compared with the cities in the south. The simplified model performed similarly to the full model, suggesting some variables were not important in explaining the genetic distance. Beyond the human population size, also the other demographic variables (population and cattle density) were retained. Conversely, only five network-related variables were retained, three for the cattle movement network (out of eight) and two for the spatial network (out of six). All network-related variables had a positive effect on the SNP distance, with the number of cattle moved in or out of a subdivision (i.e., strength), having the higher predictive effect. Interestingly, this result was similar to other studies where cattle movements alone could not fully capture *M. bovis* genetic diversity (54, 55).

### 4.4. Limitations

The major limitation of this dataset was the short data collection time window, less than a year and a half, which resulted in uncertainty in the MRCA estimate and a weak temporal signal. While we can speculate the sampled bacterial population already reached the entire study area before the 1970s, a wider sampling time window would likely allow a stronger temporal signal and improve our estimate of the MRCA, which might be prior with respect to the current estimate. In turn, this affected the pathogen's expansion patterns, including the branch velocity and wavefront, which are also limited by the sampled area size. The spatial uncertainty might also be affected by the absence of dense cattle movement records, so the known spatial coordinates associated with each sequence correspond to the last village the animal lived in. The Adamawa and Northwest regions are home to 1.25 million and 450.000 cattle, respectively (22), and while this abattoir-based study provides a very informative snapshot of the *M. bovis* population in North Cameroon, it adds to the calls to improve cattle records and movement routine data collections in LMICs (79), as well as bTB detection efforts.

The low-quality WGSs disproportionally affected the Northwest region, as presented in Supplementary Table 7. This could have hampered the representativeness of the *M. bovis* diversity in that region, reducing the number of clades observed. The Adamawa region was the most represented, despite most of the sequences excluded from the quantitative analysis because of missing coordinates, which came from the Ngaoundere abattoir. The bacterium diversity in the Northwest might also be affected by the demographic of the slaughtered cattle in the region (13) because the region is highly populated by humans and more isolated in the trade network (22), and local animals of both sexes and at any age are slaughtered. Conversely, young male calves from the Adamawa, North, and Extreme North regions are often sent to richer southern regions to maximize their economic values, leaving the older cows to be slaughtered. By being exposed to the *M. bovis* for longer, the latter has more chances to develop lesions. On the contrary, these trends likely reduce the impact of missing information on the previous location of the animals because these animals have more chances of being reared locally.

In agreement with many studies and with the *vSNP* analysis result, we used AF2122/97 as a reference genome (49, 50, 54, 55, 77, 80, 81). To account for genes, absent in *M. bovis*, Loiseau et al. (39) used *M. tuberculosis* H37Rv, a choice driven by the different purpose of their study compared with ours (define the origin and the global population structure of *M. bovis*). Generally, the pipelines used to call the SNPs differed in many of the aforementioned studies, contributing to the uncertainty of the estimates and potentially generating biases in the analysis results and the clock rate calculations.

## 5. Conclusion

In conclusion, our study indicates endemic stability of *M. bovis* is unlikely in North Cameroon, but rather the disease is slowly expanding over time. Our findings highlight the importance of collecting data in underrepresented areas to enrich insights into the current body of literature, predominantly from developed countries. Moreover, our results pave the way for future research aimed to understand whether the observed *M. bovis* high-genetic diversity affects the spread dynamics.

Our findings underscore the need to adopt a One-Health surveillance strategy for *M. bovis* control (11). More studies on combining tools such as phylogeography, statistical modeling, landscape, and ecology will be beneficial to map spread patterns and effectively inform control and preparedness strategies (54).

## Data availability statement

The Mycobacterium bovis sequences for this study have been deposited in the European Nucleotide Archive (ENA) at EMBL-EBI under accession number PRJEB61415.

## Author contributions

BB, LN, and VT conceived the original project. BB, RK, LN, VT, MS, and NE designed the field study, databases, and survey instrument. RK, NE, VT, and BB developed the field SOPs and collected the data. PM collected the cattle movement data. GR, AM, FD, and FE cleaned the data. GR, BB, SL, and AM conceived the quantitative analysis. GR and SL run the phylogenetic analysis. BS implemented the bioinformatical pipelines. GR performed the machine learning analysis and responsible for writing the initial drafts. All authors contributed to comments for the final draft, contributed to the article, and approved the submitted version.
