# Supplementary material for: Unraveling the epidemiology of Mycobacterium bovis using whole-genome sequencing combined with environmental and demographic data
Source: Front Vet Sci. 2023 May 17;10:1086001. doi: 10.3389/fvets.2023.1086001 (PMC10230100; doi:10.3389/fvets.2023.1086001)
Supplement: Supplementary file 1 [file Data_Sheet_1.pdf]

Unravelling the epidemiology of Mycobacterium bovis using whole genome sequencing combined with environmental and demographic data

Gianluigi Rossi, Barbara B. Shih, Nkongho F. Egbe, Paolo Motta, Florian Duchatel, Robert F. Kelly, Lucy Ndip, Melissa Sander, Vincent N. Tanya, Samantha J. Lycett, Barend M. Bronsvoort, Adrian Muwonge

Supplementary tables

Abattoir	Bamenda	Ngaundere	Garoua	Maroua	Total
Region	North-West	Adamawa	North	Extreme-North	-
Total examined cattle	1129	935	160	122	2346
Cultured samples from cattle w/lesions	45	106	38	18	207
Cultured samples from cattle w/o lesions	91	88	0	0	179
M. bovis positive from cattle w/lesions	31	69	34	16	150
M. bovis positive without from cattle w/o lesions	0	3	0	0	3
Total *M. bovis* positive cattle	31	72	34	16	153
Raw *M. bovis* prevalence (%)	2.75	7.7	21.25	13.11	6.52

Table S1: Number of cattle sampled in the study and number of *M. bovis* positive cattle as reported by Egbe et al. (2016, 2017, see main text for full references).

Source	ID	BioSample	Genome Name	Year	Country	Region	Host	Literature reference
Patric	1765.422	SAMN09206583	Mycobacterium tuberculosis variant bovis strain MBE9	2014	Egypt	Northern Africa	*Bos taurus* (cattle)	1
Patric	1765.423	SAMN09206586	Mycobacterium tuberculosis variant bovis strain MBE13	2014	Egypt	Northern Africa	*Bos taurus* (cattle)	1
Patric	1765.424	SAMN09206582	Mycobacterium tuberculosis variant bovis strain MBE7	2014	Egypt	Northern Africa	*Bos taurus* (cattle)	1
Patric	1765.425	SAMN09206584	Mycobacterium tuberculosis variant bovis strain MBE10	2014	Egypt	Northern Africa	*Bos taurus* (cattle)	1
Patric	1765.426	SAMN09206585	Mycobacterium tuberculosis variant bovis strain MBE12	2014	Egypt	Northern Africa	*Bos taurus* (cattle)	1
Patric	1765.427	SAMN09206581	Mycobacterium tuberculosis variant bovis strain MBE6	2014	Egypt	Northern Africa	*Bos taurus* (cattle)	1
Patric	1765.428	SAMN09206580	Mycobacterium tuberculosis variant bovis strain MBE5	2017	Egypt	Northern Africa	*Bos taurus* (cattle)	1
Patric	1765.429	SAMN09206579	Mycobacterium tuberculosis variant bovis strain MBE4	2015	Egypt	Northern Africa	*Bos taurus* (cattle)	1
Patric	1765.43	SAMN09206578	Mycobacterium tuberculosis variant bovis strain MBE3	2014	Egypt	Northern Africa	*Bos taurus* (cattle)	1
Patric	1765.431	SAMN09206577	Mycobacterium tuberculosis variant bovis strain MBE2	2017	Egypt	Northern Africa	*Bos taurus* (cattle)	1
Patric	1765.432	SAMN09206576	Mycobacterium tuberculosis variant bovis strain MBE1	2014	Egypt	Northern Africa	*Bos taurus* (cattle)	1
Patric	33892.41	SAMN10279424	Mycobacterium tuberculosis variant bovis BCG strain BCG-S48	2015	South Africa	Southern Africa	*Homo sapiens* (human)	1
Patric	33892.42	SAMN10278076	Mycobacterium tuberculosis variant bovis BCG strain BCG_S49	2014	South Africa	Southern Africa	*Homo sapiens* (human)	1
Patric	1354193.3	SAMN02231112	Mycobacterium bovis MAL010093	NA	Mali	Western Africa	*Homo sapiens* (human)	1
Patric	1438822.3	SAMN02567762	Mycobacterium bovis B2 7505	2001	Uganda	Eastern Africa	*Homo sapiens* (human)	1
Patric	1438823.3	SAMN02567761	Mycobacterium bovis Bz 31150	2007	Uganda	Eastern Africa	*Homo sapiens* (human)	1
Patric	1438824.3	SAMN02567763	Mycobacterium bovis D 4155	2008	Uganda	Eastern Africa	*Homo sapiens* (human)	1
Patric	1438825.3	SAMN02567764	Mycobacterium bovis Kc 32216	2004	Uganda	Eastern Africa	*Homo sapiens* (human)	1
Patric	1438826.3	SAMN02567767	Mycobacterium bovis Kc 9614	2006	Uganda	Eastern Africa	*Homo sapiens* (human)	1
Patric	1438827.3	SAMN02567766	Mycobacterium bovis Mr 4387	2006	Uganda	Eastern Africa	*Homo sapiens* (human)	1
Patric	1438828.3	SAMN02567760	Mycobacterium bovis Wt 21231	2006	Uganda	Eastern Africa	*Homo sapiens* (human)	1

Source	ID	BioSample	Genome Name	Year	Country	Region	Host	Literature reference
Patric	1438829.3	SAMN02567765	Mycobacterium bovis Wt 21419	2001	Uganda	Eastern Africa	*Homo sapiens* (human)	1
EBI	ERR1203064	SAMEA3504611	Mycobacterium tuberculosis variant bovis	2014	Ghana	Western Africa	*Homo sapiens* (human)	2
EBI	ERR2659159	SAMEA4752945	Mycobacterium tuberculosis variant bovis	2005	Nigeria	Western Africa	NA	STPHI, Switzerland
EBI	ERR2659160	SAMEA4752945	Mycobacterium tuberculosis variant bovis	2005	Nigeria	Western Africa	NA	STPHI, Switzerland
EBI	ERR502499	SAMEA2340906	Mycobacterium tuberculosis variant bovis	2011	Ghana	Western Africa	*Homo sapiens* (human)	2
EBI	ERR502526	SAMEA2340934	Mycobacterium tuberculosis variant bovis	2012	Ghana	Western Africa	*Homo sapiens* (human)	2
EBI	ERR502529	SAMEA2340937	Mycobacterium tuberculosis variant bovis	2012	Ghana	Western Africa	*Homo sapiens* (human)	2
EBI	ERR502538	SAMEA2340948	Mycobacterium tuberculosis variant bovis	2012	Ghana	Western Africa	*Homo sapiens* (human)	2
EBI	ERS1028697	SAMEA3721548	Mycobacterium tuberculosis variant bovis	NA	Ethiopia	Eastern Africa	NA	3
EBI	ERS107856	SAMEA1485230	Mycobacterium tuberculosis variant bovis	2003	Malawi	Eastern Africa	NA	4
EBI	ERS108068	SAMEA1485268	Mycobacterium tuberculosis variant bovis	2003	Malawi	Eastern Africa	BCG	4
EBI	ERS142115	SAMEA1556837	Mycobacterium tuberculosis variant bovis	2005	Malawi	Eastern Africa	NA	4
EBI	ERS1534748	SAMEA72475168	Mycobacterium tuberculosis variant bovis	1998	South Africa	Southern Africa	*Panthera leo* (lion)	5
EBI	ERS1534750	SAMEA72476668	Mycobacterium tuberculosis variant bovis	1997	South Africa	Southern Africa	*Syncerus caffer* (buffalo)	5
EBI	ERS1534751	SAMEA72477418	Mycobacterium tuberculosis variant bovis	1997	South Africa	Southern Africa	*Tragelaphus strepsiceros* (kudu)	5
EBI	ERS1534752	SAMEA72478168	Mycobacterium tuberculosis variant bovis	1999	South Africa	Southern Africa	*Syncerus caffer* (buffalo)	5
EBI	ERS1534753	SAMEA72478918	Mycobacterium tuberculosis variant bovis	1998	South Africa	Southern Africa	*Syncerus caffer* (buffalo)	5
EBI	ERS1534754	SAMEA72479668	Mycobacterium tuberculosis variant bovis	1999	South Africa	Southern Africa	*Syncerus caffer* (buffalo)	5
EBI	ERS1534757	SAMEA72481918	Mycobacterium tuberculosis variant bovis	2003	South Africa	Southern Africa	*Bos taurus* (cattle)	5
EBI	ERS1534758	SAMEA72482668	Mycobacterium tuberculosis variant bovis	1998	South Africa	Southern Africa	*Syncerus caffer* (buffalo)	5
EBI	ERS1534759	SAMEA72483418	Mycobacterium tuberculosis variant bovis	1996	South Africa	Southern Africa	*Syncerus caffer* (buffalo)	5
EBI	ERS1534761	SAMEA72484918	Mycobacterium tuberculosis variant bovis	2000	South Africa	Southern Africa	*Syncerus caffer* (buffalo)	5

Source	ID	BioSample	Genome Name	Year	Country	Region	Host	Literature reference
EBI	ERS1534762	SAMEA72485668	Mycobacterium tuberculosis variant bovis	2000	South Africa	Southern Africa	*Bos taurus* (cattle)	5
EBI	ERS1534763	SAMEA72486418	Mycobacterium tuberculosis variant bovis	1999	South Africa	Southern Africa	*Panthera leo* (lion)	5
EBI	ERS1534764	SAMEA72487168	Mycobacterium tuberculosis variant bovis	1998	South Africa	Southern Africa	*Panthera leo* (lion)	5
EBI	ERS153903	SAMEA1569110	Mycobacterium tuberculosis variant bovis	2004	Malawi	Eastern Africa	BCG	4
EBI	ERS153942	SAMEA1569365	Mycobacterium tuberculosis variant bovis	1999	Malawi	Eastern Africa	*Homo sapiens* (human)	4
EBI	ERS2400060	SAMEA3176565	Mycobacterium tuberculosis variant bovis	NA	South Africa	Southern Africa	NA	CTB (NICD), SAF
EBI	ERS3607829	SAMEA5819237	Mycobacterium tuberculosis variant bovis	2007	Ethiopia	Eastern Africa	*Bos taurus* (cattle)	6
EBI	ERS3607830	SAMEA5819238	Mycobacterium tuberculosis variant bovis	2009	Ethiopia	Eastern Africa	*Homo sapiens* (human)	6
EBI	ERS3607831	SAMEA5819239	Mycobacterium tuberculosis variant bovis	2009	Ethiopia	Eastern Africa	*Homo sapiens* (human)	6
EBI	ERS3607832	SAMEA5819240	Mycobacterium tuberculosis variant bovis	2010	Ethiopia	Eastern Africa	*Homo sapiens* (human)	6
EBI	ERS3607833	SAMEA5819241	Mycobacterium tuberculosis variant bovis	2007	Ethiopia	Eastern Africa	*Bos taurus* (cattle)	6
EBI	ERS3607834	SAMEA5819242	Mycobacterium tuberculosis variant bovis	2008	Ethiopia	Eastern Africa	*Bos taurus* (cattle)	6
EBI	ERS3607835	SAMEA5819243	Mycobacterium tuberculosis variant bovis	2007	Ethiopia	Eastern Africa	*Bos taurus* (cattle)	6
EBI	ERS3607836	SAMEA5819244	Mycobacterium tuberculosis variant bovis	1993	Burundi	Eastern Africa	*Bos taurus* (cattle)	6
EBI	SRS1237685	SAMN04385646	Mycobacterium tuberculosis variant bovis	2011	Tunisia	Northern Africa	*Homo sapiens* (human)	7
EBI	SRS2152613	SAMN06847536	Mycobacterium tuberculosis variant bovis	NA	Ethiopia	Eastern Africa	*Bos taurus* (cattle)	USDA, USA
EBI	SRS2152614	SAMN06847535	Mycobacterium tuberculosis variant bovis	NA	Ethiopia	Eastern Africa	*Bos taurus* (cattle)	USDA, USA
EBI	SRS2152615	SAMN06847550	Mycobacterium tuberculosis variant bovis	NA	Eritrea	Eastern Africa	*Bos taurus* (cattle)	USDA, USA
EBI	SRS2152616	SAMN06847549	Mycobacterium tuberculosis variant bovis	NA	Eritrea	Eastern Africa	*Bos taurus* (cattle)	USDA, USA
EBI	SRS2152617	SAMN06847548	Mycobacterium tuberculosis variant bovis	NA	Eritrea	Eastern Africa	*Bos taurus* (cattle)	USDA, USA
EBI	SRS2152618	SAMN06847547	Mycobacterium tuberculosis variant bovis	NA	Eritrea	Eastern Africa	*Bos taurus* (cattle)	USDA, USA
EBI	SRS2152619	SAMN06847546	Mycobacterium tuberculosis variant bovis	NA	Eritrea	Eastern Africa	*Bos taurus* (cattle)	USDA, USA

Source	ID	BioSample	Genome Name	Year	Country	Region	Host	Literature reference
EBI	SRS2152620	SAMN06847545	Mycobacterium tuberculosis variant bovis	NA	Eritrea	Eastern Africa	*Bos taurus* (cattle)	USDA, USA
EBI	SRS2152621	SAMN06847544	Mycobacterium tuberculosis variant bovis	NA	Eritrea	Eastern Africa	*Bos taurus* (cattle)	USDA, USA
EBI	SRS2152622	SAMN06847543	Mycobacterium tuberculosis variant bovis	NA	Eritrea	Eastern Africa	*Bos taurus* (cattle)	USDA, USA
EBI	SRS2152623	SAMN06847542	Mycobacterium tuberculosis variant bovis	NA	Eritrea	Eastern Africa	*Bos taurus* (cattle)	USDA, USA
EBI	SRS2152624	SAMN06847541	Mycobacterium tuberculosis variant bovis	NA	Eritrea	Eastern Africa	*Bos taurus* (cattle)	USDA, USA
EBI	SRS2152625	SAMN06847540	Mycobacterium tuberculosis variant bovis	NA	Eritrea	Eastern Africa	*Bos taurus* (cattle)	USDA, USA
EBI	SRS2152626	SAMN06847539	Mycobacterium tuberculosis variant bovis	NA	Eritrea	Eastern Africa	*Bos taurus* (cattle)	USDA, USA
EBI	SRS2152627	SAMN06847538	Mycobacterium tuberculosis variant bovis	NA	Eritrea	Eastern Africa	*Bos taurus* (cattle)	USDA, USA
EBI	SRS2152628	SAMN06847537	Mycobacterium tuberculosis variant bovis	NA	Eritrea	Eastern Africa	*Bos taurus* (cattle)	USDA, USA
EBI	SRS3269271	SAMN09090520	Mycobacterium tuberculosis variant bovis	2016	Morocco	Northern Africa	*Homo sapiens* (human)	7
EBI	SRS3939513	SAMN10247369	Mycobacterium tuberculosis variant bovis	NA	Ethiopia	Eastern Africa	*Bos taurus* (cattle)	USDA, USA
EBI	SRS3939514	SAMN10247365	Mycobacterium tuberculosis variant bovis	NA	Ethiopia	Eastern Africa	*Bos taurus* (cattle)	USDA, USA
EBI	SRS3939515	SAMN10247363	Mycobacterium tuberculosis variant bovis	NA	Ethiopia	Eastern Africa	*Bos taurus* (cattle)	USDA, USA
EBI	SRS3939516	SAMN10247366	Mycobacterium tuberculosis variant bovis	NA	Ethiopia	Eastern Africa	*Bos taurus* (cattle)	USDA, USA
EBI	SRS3939517	SAMN10247371	Mycobacterium tuberculosis variant bovis	NA	Ethiopia	Eastern Africa	*Bos taurus* (cattle)	USDA, USA
EBI	SRS3939518	SAMN10247368	Mycobacterium tuberculosis variant bovis	NA	Ethiopia	Eastern Africa	*Bos taurus* (cattle)	USDA, USA
EBI	SRS3939519	SAMN10247357	Mycobacterium tuberculosis variant bovis	NA	Ethiopia	Eastern Africa	*Bos taurus* (cattle)	USDA, USA
EBI	SRS3939520	SAMN10247367	Mycobacterium tuberculosis variant bovis	NA	Ethiopia	Eastern Africa	*Bos taurus* (cattle)	USDA, USA
EBI	SRS3939521	SAMN10247360	Mycobacterium tuberculosis variant bovis	NA	Ethiopia	Eastern Africa	*Bos taurus* (cattle)	USDA, USA
EBI	SRS3939522	SAMN10247361	Mycobacterium tuberculosis variant bovis	NA	Ethiopia	Eastern Africa	*Bos taurus* (cattle)	USDA, USA
EBI	SRS3939523	SAMN10247359	Mycobacterium tuberculosis variant bovis	NA	Ethiopia	Eastern Africa	*Bos taurus* (cattle)	USDA, USA
EBI	SRS3939524	SAMN10247372	Mycobacterium tuberculosis variant bovis	NA	Ethiopia	Eastern Africa	*Bos taurus* (cattle)	USDA, USA

Source	ID	BioSample	Genome Name	Year	Country	Region	Host	Literature reference
EBI	SRS3939525	SAMN10247362	Mycobacterium tuberculosis variant bovis	NA	Ethiopia	Eastern Africa	*Bos taurus* (cattle)	USDA, USA
EBI	SRS3939526	SAMN10247364	Mycobacterium tuberculosis variant bovis	NA	Ethiopia	Eastern Africa	*Bos taurus* (cattle)	USDA, USA
EBI	SRS3939527	SAMN10247370	Mycobacterium tuberculosis variant bovis	NA	Ethiopia	Eastern Africa	*Bos taurus* (cattle)	USDA, USA
EBI	SRS3939528	SAMN10247358	Mycobacterium tuberculosis variant bovis	NA	Ethiopia	Eastern Africa	*Bos taurus* (cattle)	USDA, USA
EBI	SRS3940198	SAMN10247730	Mycobacterium tuberculosis variant bovis	NA	Zambia	Eastern Africa	*Bos taurus* (cattle)	USDA, USA
EBI	SRS3940199	SAMN10247733	Mycobacterium tuberculosis variant bovis	NA	Zambia	Eastern Africa	*Bos taurus* (cattle)	USDA, USA
EBI	SRS3940200	SAMN10247731	Mycobacterium tuberculosis variant bovis	NA	Zambia	Eastern Africa	*Bos taurus* (cattle)	USDA, USA
EBI	SRS3940201	SAMN10247732	Mycobacterium tuberculosis variant bovis	NA	Zambia	Eastern Africa	*Bos taurus* (cattle)	USDA, USA
EBI	SRS3940202	SAMN10247735	Mycobacterium tuberculosis variant bovis	NA	Zambia	Eastern Africa	*Bos taurus* (cattle)	USDA, USA
EBI	SRS3940203	SAMN10247734	Mycobacterium tuberculosis variant bovis	NA	Zambia	Eastern Africa	*Bos taurus* (cattle)	USDA, USA
EBI	SRS3940204	SAMN10247737	Mycobacterium tuberculosis variant bovis	NA	Zambia	Eastern Africa	*Bos taurus* (cattle)	USDA, USA
EBI	SRS3940205	SAMN10247736	Mycobacterium tuberculosis variant bovis	NA	Zambia	Eastern Africa	*Bos taurus* (cattle)	USDA, USA
EBI	SRS3940412	SAMN10247637	Mycobacterium tuberculosis variant bovis	NA	Uganda	Eastern Africa	*Bos taurus* (cattle)	USDA, USA
EBI	SRS3940413	SAMN10247635	Mycobacterium tuberculosis variant bovis	NA	Uganda	Eastern Africa	*Bos taurus* (cattle)	USDA, USA
EBI	SRS3940414	SAMN10247634	Mycobacterium tuberculosis variant bovis	NA	Uganda	Eastern Africa	*Bos taurus* (cattle)	USDA, USA
EBI	SRS3940415	SAMN10247633	Mycobacterium tuberculosis variant bovis	NA	Uganda	Eastern Africa	*Bos taurus* (cattle)	USDA, USA
EBI	SRS3940416	SAMN10247636	Mycobacterium tuberculosis variant bovis	NA	Uganda	Eastern Africa	*Bos taurus* (cattle)	USDA, USA
EBI	SRS3940417	SAMN10247629	Mycobacterium tuberculosis variant bovis	NA	Uganda	Eastern Africa	*Bos taurus* (cattle)	USDA, USA
EBI	SRS3940418	SAMN10247630	Mycobacterium tuberculosis variant bovis	NA	Uganda	Eastern Africa	*Bos taurus* (cattle)	USDA, USA
EBI	SRS3940419	SAMN10247631	Mycobacterium tuberculosis variant bovis	NA	Uganda	Eastern Africa	*Bos taurus* (cattle)	USDA, USA
EBI	SRS3940420	SAMN10247632	Mycobacterium tuberculosis variant bovis	NA	Uganda	Eastern Africa	*Bos taurus* (cattle)	USDA, USA
EBI	SRS3940421	SAMN10247625	Mycobacterium tuberculosis variant bovis	NA	Uganda	Eastern Africa	*Bos taurus* (cattle)	USDA, USA

Source	ID	BioSample	Genome Name	Year	Country	Region	Host	Literature reference
EBI	SRS3940422	SAMN10247626	Mycobacterium tuberculosis variant bovis	NA	Uganda	Eastern Africa	*Bos taurus* (cattle)	USDA, USA
EBI	SRS3940423	SAMN10247623	Mycobacterium tuberculosis variant bovis	NA	Uganda	Eastern Africa	*Bos taurus* (cattle)	USDA, USA
EBI	SRS3940424	SAMN10247624	Mycobacterium tuberculosis variant bovis	NA	Uganda	Eastern Africa	*Bos taurus* (cattle)	USDA, USA
EBI	SRS3940425	SAMN10247621	Mycobacterium tuberculosis variant bovis	NA	Uganda	Eastern Africa	*Bos taurus* (cattle)	USDA, USA
EBI	SRS3940426	SAMN10247619	Mycobacterium tuberculosis variant bovis	NA	Uganda	Eastern Africa	*Bos taurus* (cattle)	USDA, USA
EBI	SRS3940427	SAMN10247620	Mycobacterium tuberculosis variant bovis	NA	Uganda	Eastern Africa	*Bos taurus* (cattle)	USDA, USA
EBI	SRS3940428	SAMN10247628	Mycobacterium tuberculosis variant bovis	NA	Uganda	Eastern Africa	*Bos taurus* (cattle)	USDA, USA
EBI	SRS3940429	SAMN10247622	Mycobacterium tuberculosis variant bovis	NA	Uganda	Eastern Africa	*Bos taurus* (cattle)	USDA, USA
EBI	SRS3940430	SAMN10247627	Mycobacterium tuberculosis variant bovis	NA	Uganda	Eastern Africa	*Bos taurus* (cattle)	USDA, USA
EBI	SRS560022	SAMN02567761	Mycobacterium tuberculosis variant bovis Bz 31150	2007	Uganda	Eastern Africa	Primate (chimpanzee)	8
EBI	SRS839036	SAMN03300068	Mycobacterium tuberculosis variant bovis	2001	Tanzania	Eastern Africa	Primate (unknown)	USDA, USA
EBI	SRS839042	SAMN03300070	Mycobacterium tuberculosis variant bovis	2001	Tanzania	Eastern Africa	Primate (unknown)	USDA, USA

Table S2: Public repositories *M. bovis* whole-genome sequences (WGS) isolates used in this study to generate an African phylogenetic tree.

References (in case of no literature reference on GenBank the institute submitting the sequence was reported instead):

1. Wattam, A. R. *et al.* (2017) 'Improvements to PATRIC, the all-bacterial bioinformatics database and analysis resource center', *Nucleic Acids Research*, 45(D1), pp. D535–D542. doi: 10.1093/nar/gkw1017;

2. Otchere, I. D. *et al.* (2019) 'Molecular epidemiology and whole genome sequencing analysis of clinical *Mycobacterium bovis* from Ghana', *PLoS ONE*, 14(3), pp. 1–13. doi: 10.1371/journal.pone.0209395;

3. Mekonnen, D. *et al.* (2019) 'Molecular epidemiology of *M. tuberculosis* in Ethiopia: A systematic review and meta-analysis', *Tuberculosis*, 118(June). doi: 10.1016/j.tube.2019.101858;

4. Guerra-Assunçaõ, J. A. *et al.* (2015) 'Recurrence due to relapse or reinfection with *Mycobacterium tuberculosis*: A whole-genome sequencing approach in a large, population-based cohort with a high HIV infection prevalence and active follow-up', *Journal of Infectious Diseases*, 211(7), pp. 1154–1163. doi: 10.1093/infdis/jiu574;

5. Dippenaar, A. *et al.* (2017) 'Progenitor strain introduction of *Mycobacterium bovis* at the wildlife-livestock interface can lead to clonal expansion of the disease in a single ecosystem', *Infection, Genetics and Evolution*, 51, pp. 235–238. doi: 10.1016/j.meegid.2017.04.012;

6. Loiseau, C. *et al.* (2020) 'An African origin for Mycobacterium bovis', *Evolution, Medicine and Public Health*, 2020(1), pp. 49–59. doi: 10.1093/EMPH/EOAA005;

7. Schena, E. *et al.* (2016) 'Delamanid susceptibility testing of *Mycobacterium tuberculosis* using the resazurin microtitre assay and the BACTEC™ MGIT™ 960 system', *Journal of Antimicrobial Chemotherapy*, 71(6), pp. 1532–1539. doi: 10.1093/jac/dkw044;

8. Wanzala, S. I. *et al.* (2015) 'Draft genome sequences of *Mycobacterium bovis* BZ 31150 and *Mycobacterium bovis* B2 7505, pathogenic bacteria isolated from archived captive animal bronchial washes and human sputum samples in Uganda', *Genome Announcements*, 3(5), pp. 14–15. doi: 10.1128/genomeA.01102-15.

Source	Dataset	Link	Selected variable(s)	Year
FAO Map Catalog	Model of cattle density (GLW 2.01)	https://data.apps.fao.org/	Cattle density	2010
The Humanitarian Data Exchange	OCHA West and Central Africa, digital elevation model	https://data.humdata.org/	Elevation	NA
World Pop Hub	Population density	https://hub.worldpop.org/	Human density	2013
European Space Agency	Global land cover maps	https://www.esa.int/	Forest %	2015
			Waterbodies %	
			Cropland and Grassland %	
			Mosaic, shrub and other vegetation %	
Geofrabrik	Open street map data extracts	http://download.geofabrik.de	Roads length	NA
			Number of intersections	

Table S3: Description and sources of the geographical variables' raster used in the *seraphim* model. When multiple updates were available, we chose the one closest to the data collection campaign in 2012 and 2013.

Joined raster	Code	Original description
Cropland-grassland	10	Cropland rainfed
	20	Cropland irrigated or post-flooding
	30	Mosaic cropland (>50%) / natural vegetation (tree shrub herbaceous cover) (<50%)
	130	Grassland
	11	Herbaceous cover
Forest	50	Tree cover broadleaved evergreen closed to open (>15%)
	60	Tree cover broadleaved deciduous closed to open (>15%)
	61	Tree cover broadleaved deciduous closed (>40%)
	62	Tree cover broadleaved deciduous open (15-40%)
	70	Tree cover needleleaved evergreen closed to open (>15%)
	71	Tree cover needleleaved evergreen closed (>40%)
	72	Tree cover needleleaved evergreen open (15-40%)
	80	Tree cover needleleaved deciduous closed to open (>15%)
	81	Tree cover needleleaved deciduous closed (>40%)
	82	Tree cover needleleaved deciduous open (15-40%)
	90	Tree cover mixed leaf type (broadleaved and needleleaved)
Mosaic_shrub_otherv	12	Tree or shrub cover
	40	Mosaic natural vegetation (tree shrub herbaceous cover) (>50%) / cropland (<50%)
	100	Mosaic tree and shrub (>50%) / herbaceous cover (<50%)
	110	Mosaic herbaceous cover (>50%) / tree and shrub (<50%)
	120	Shrubland
	121	Shrubland evergreen
	122	Shrubland deciduous
	140	Lichens and mosses
	150	Sparse vegetation (tree shrub herbaceous cover) (<15%)
	151	Sparse tree (<15%)
	152	Sparse shrub (<15%)
	153	Sparse herbaceous cover (<15%)
	180	Shrub or herbaceous cover flooded fresh/saline/brakish water
Waterbodies	210	Water bodies
Not used	0	No data
	160	Tree cover flooded fresh or brakish water
	170	Tree cover flooded saline water
	190	Urban areas
	200	Bare areas
	201	Consolidated bare areas
	202	Unconsolidated bare areas
	220	Permanent snow and ice

Table S4: The list of land cover types included in the European Space Agency Land Cover raster (with the relative code) and the four joined raster types used in this study.

#	Variable	Type	Description	Selected by simplified model
1	TemporalDistance	Temporal	Time (days) between the two isolates sampling	YES
2	SpatDistance	Spatial	Distance (km) between the two isolates' cattle villages	YES
3	SameSubdivision	Spatial	The two isolates cattle from the same administrative subdivision (binary)	NO
4	SameSpoligotype	Molecular	The two isolates presents the same spoligotype (binary)	YES
5	SameMIRUVNTR	Molecular	The two isolates presents the same MIRU-VNTR type (binary)	YES
6	SameClade	Molecular	The two isolates are in the same clade (binary)	YES
7	PopulationDensity.x	Demographic	Population density of the oldest isolate subdivision	YES
8	PopulationDensity.y	Demographic	Population density of the youngest isolate subdivision	YES
9	Population.x	Demographic	Population of the oldest isolate subdivision	YES
10	Population.y	Demographic	Population of the youngest isolate subdivision	YES
11	CattleDensity.x	Demographic	Cattle density of the oldest isolate subdivision	YES
12	CattleDensity.y	Demographic	Cattle density of the youngest isolate subdivision	YES
13	NumMarkets.x	Demographic	Number of markets in the oldest isolate subdivision	NO
14	NumMarkets.y	Demographic	Number of markets in the youngest isolate subdivision	NO
15	SpatNet_degree.x	Network/spatial	Spatial network degree of the oldest isolate subdivision	YES
16	SpatNet_degree.y	Network/spatial	Spatial network degree of the youngest isolate subdivision	NO
17	SpatNet_betweenness.x	Network/spatial	Spatial network betweenness centrality of the oldest isolate subdivision	YES
18	SpatNet_betweenness.y	Network/spatial	Spatial network betweenness centrality of the youngest isolate subdivision	YES
19	SpatNet_shortestPath	Network/spatial	Spatial network shortest path length between the two isolates subdivisions	NO
20	SpatNet_sameCommunity	Network/spatial	The two isolates are in the same spatial network community (binary)	NO
21	MovNet_degree.x	Network/demographic	Cattle movement network degree of the oldest isolate subdivision	YES
22	MovNet_degree.y	Network/demographic	Cattle movement network degree of the youngest isolate subdivision	NO
23	MovNet_betweenness.x	Network/demographic	Cattle movement network betweenness centrality of the oldest isolate subdivision	NO
24	MovNet_betweenness.y	Network/demographic	Cattle movement network betweenness centrality of the youngest isolate subdivision	NO
25	MovNet_strength.x	Network/demographic	Cattle movement network strength of the oldest isolate subdivision	YES
26	MovNet_strength.y	Network/demographic	Cattle movement network strength of the youngest isolate subdivision	YES
27	MovNet_shortestPath	Network/demographic	Cattle movement network shortest path length between the two isolates subdivisions	NO
28	MovNet_sameCommunity	Network/demographic	The two isolates are in the same cattle movement network community (binary)	NO

Table S5: The list of covariates used in the boosted regression trees model to explain the SNP distance. All covariates are calculated between each pair of *M. bovis* isolate ("older" and "youngest" refers to the one sampled first and last among the pair).

Abattoir	Bamend a	Ngaundere	Garoua	Maroua	Total
**Abattoir's region**	**North-West**	**Adamawa**	**North**	**Extreme-North**	**-**
Total examined cattle	1'129	935	160	122	2'346
Total *M. bovis* positive cattle	31	72	34	16	153
Raw *M. bovis* prevalence (%)	2.75	7.7	21.25	13.11	6.52
WGS attempts	22	81	14	14	131
Total sequences obtained	22	72	14	14	122
Cattle sampled	21	71	13	13	118
Excluded sequences, low quality/contami nated/low coverage	16	9	2	5	32
Excluded sequences, not *M. bovis*	1	0	0	0	1
Excluded sequences, location missing	0	24*	0	0	24*
Excluded sequences, outliers	0	1*	0	1	2*
Sequences included in full analysis	5	39	12	8	64
Positive animals sequenced with full metadata and high quality WGS (%)	16.1	52.7	32.4	50.0	40.5

Table S6: Number of the available and discarded Cameroonian *M. bovis* WGS cattle isolates, divided by the abattoir they were sampled at. One sequence (marked with*) sampled in the Ngaundere abattoir is counted twice because it lacked location and was an outlier in the qualitative phylogenetic tree. Two sequences from humans were not counted in the table.

Step	Substitution model	Clock model	Population model	Spatial model	Log MLE (PS)	Log MLE (SS)
1	Hasegawa-Kishino-Yano (HKY)	Strict	Constant	Browniam random walk	-10076.78	-10065.28
1	HKY	Strict	Constant	Cauchy Relaxed Random Walk (RRW)	-10032.80	-10032.86
1	HKY	Strict	Constant	Lognormal RRW	-10042.07	-10042.29
1 -> 2	HKY	Strict	Constant	Gamma RRW	-10027.32	-10030.12
2	HKY	Strict	Exponential	Gamma RRW	-10001.33	-10003.28
2 (final)	*HKY*	*Strict*	*SkyGrid*	*Gamma RRW*	*-9662.16*	*-9663.11*

Table S7: Results of the best model selection using BEAST.

Abattoir	**Bamenda**	**Ngaundere**	**Garoua**	**Maroua**	**Total**
Region	**North-West**	**Adamawa**	**North**	**Extreme-North**	
M. bovis isolates*	5	39	12	8	64
Clade 1	2	12	6	2	22
Clade 2	3	13	1	0	17
Clade 3	0	8	5	6	19
Clade 4	0	5	0	0	5
No clade	0	1	0	0	1

Table S8: Number of *M. bovis* isolates divided by abattoir of sampling and by clade. *We counted the high-quality only included in the quantitative analyses.

Supplementary figures

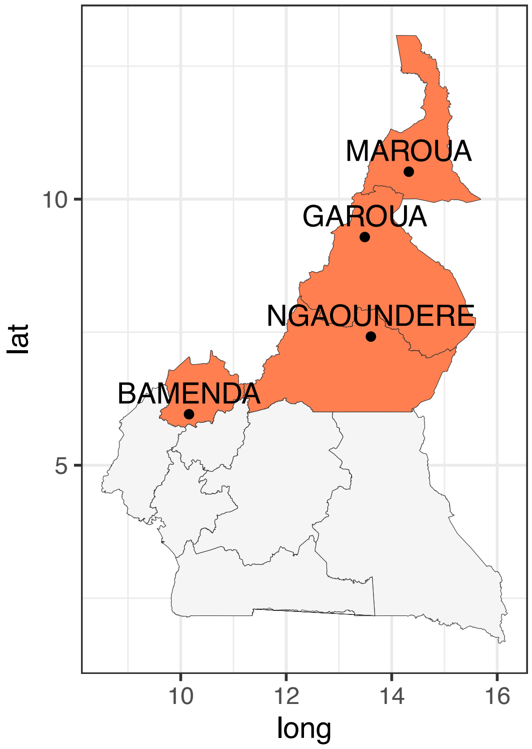

Figure S1: The location of the four regional sampled abattoirs: Bamenda (North West region), Ngaoundere (Adamawa region), Garoua (North region), and Maroua (Extreme North region).

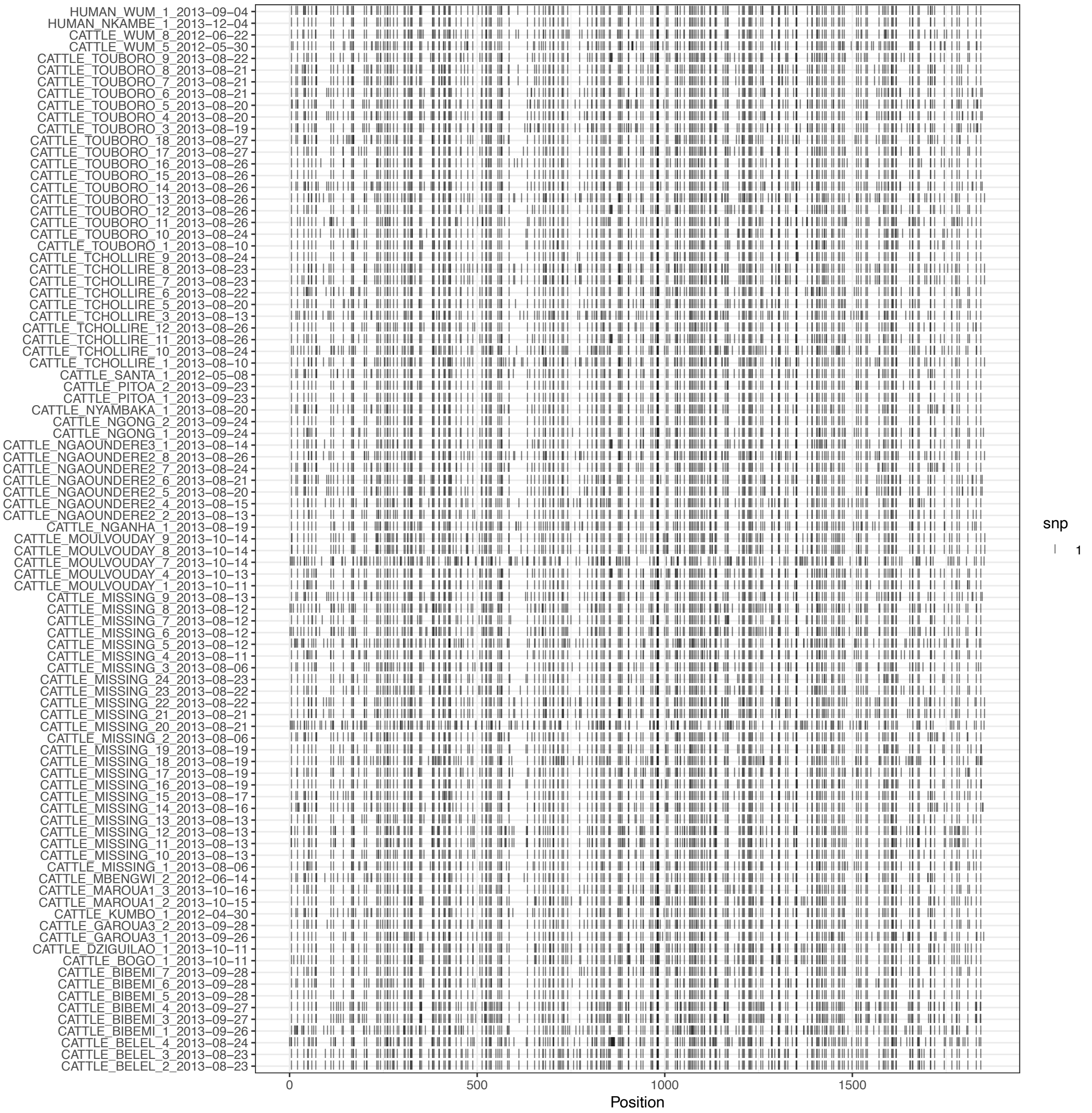

Figure S2: Complete SNPs of the 91 high-quality Cameroonian *M. bovis* sequences. All sequences have been deposited in the European Nucleotide Archive (ENA) at EMBL-EBI, accession number PRJEB61415 (https://www.ebi.ac.uk/ena/browser/view/PRJEB61415).

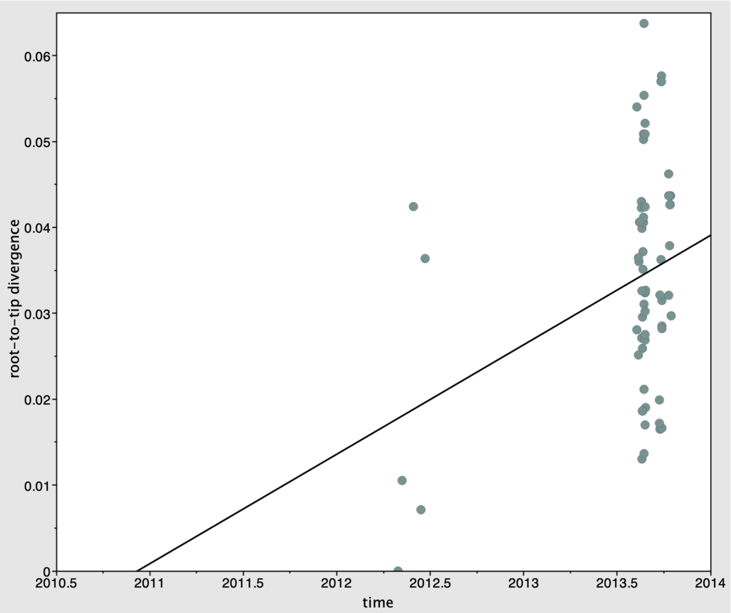

Figure S3: the root-to-tip distance plotted against the temporal distance for the 64 high-quality Cameroonian sequences.

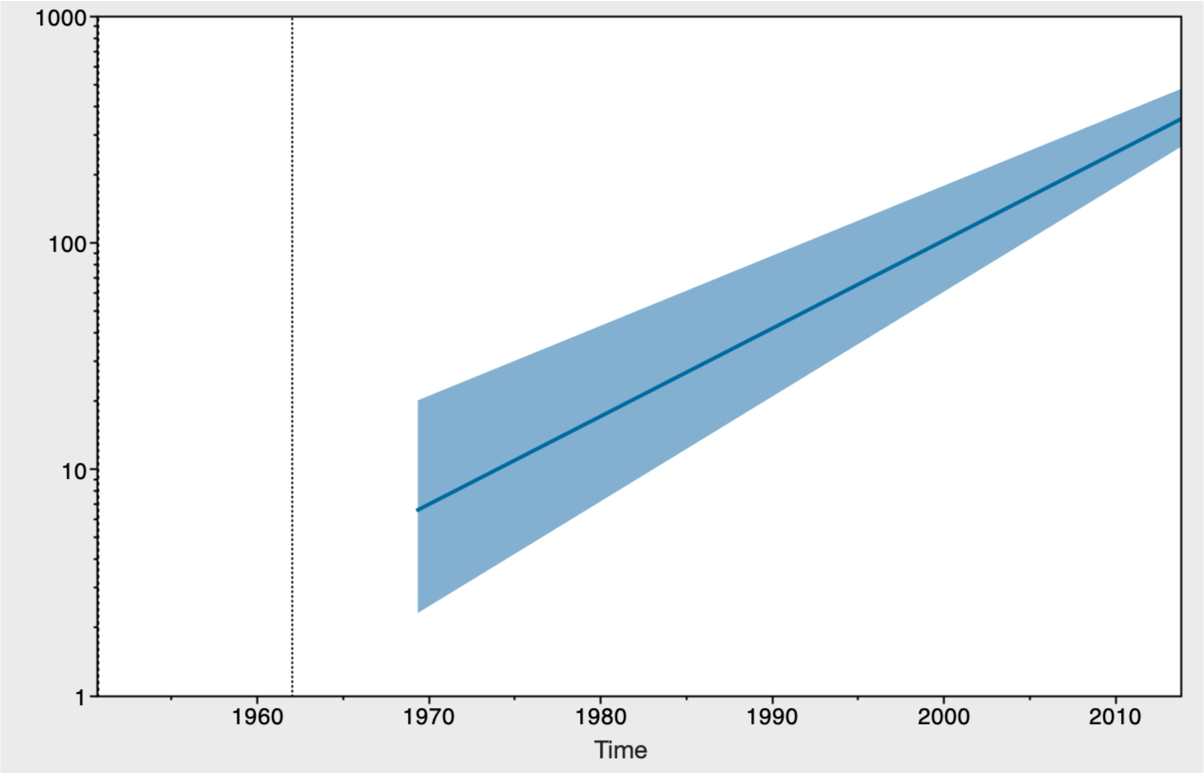

Figure S4: The *SkyGrid* estimates of the effective population growth in BEAST.

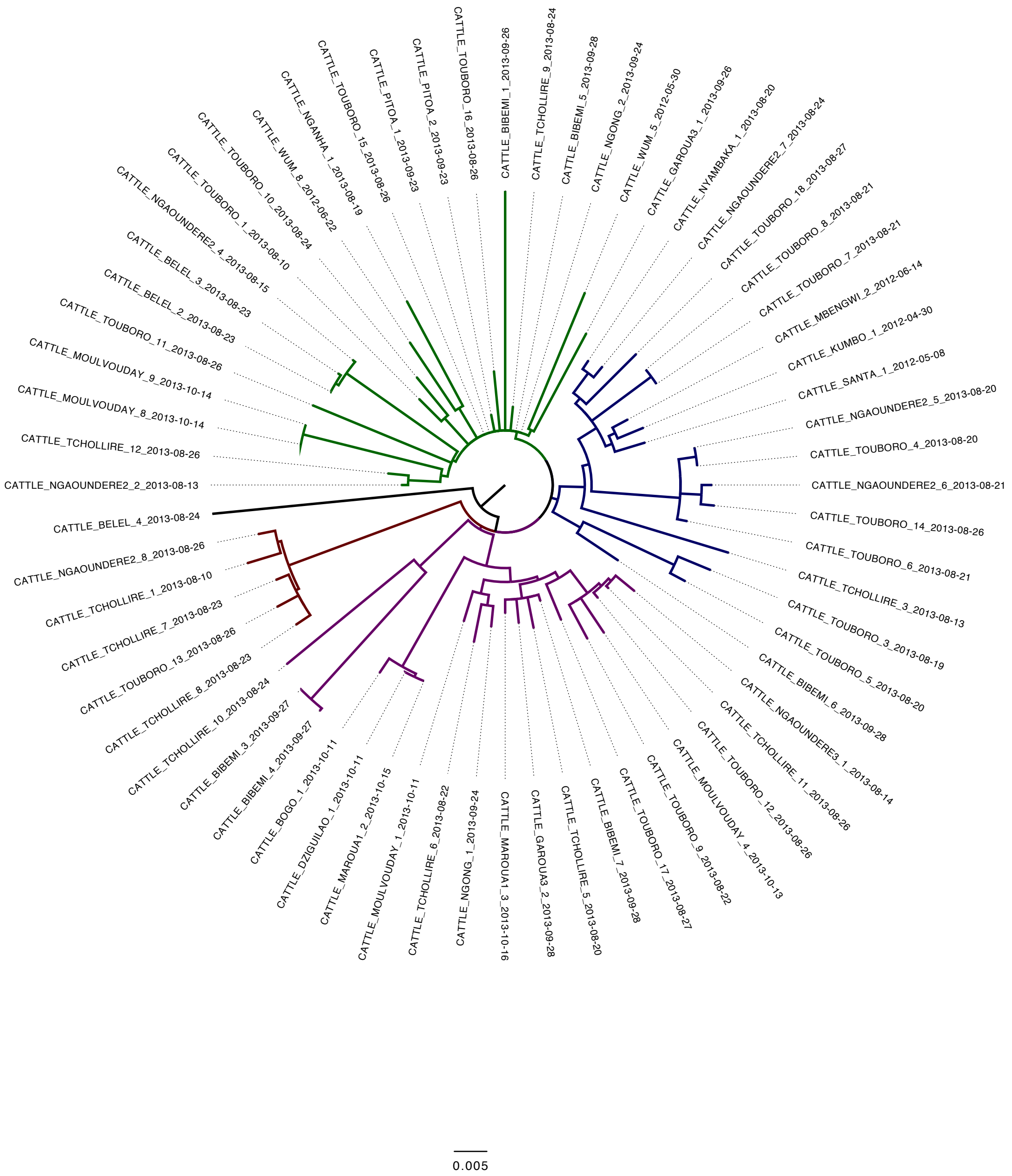

Figure S5: Phylogenetic maximum-likelihood tree of the 64 high-quality Cameroonian *M. bovis* sequences showing the genetic distance. The tree was obtained with *iqtree* software and plotted with *FigTree*.

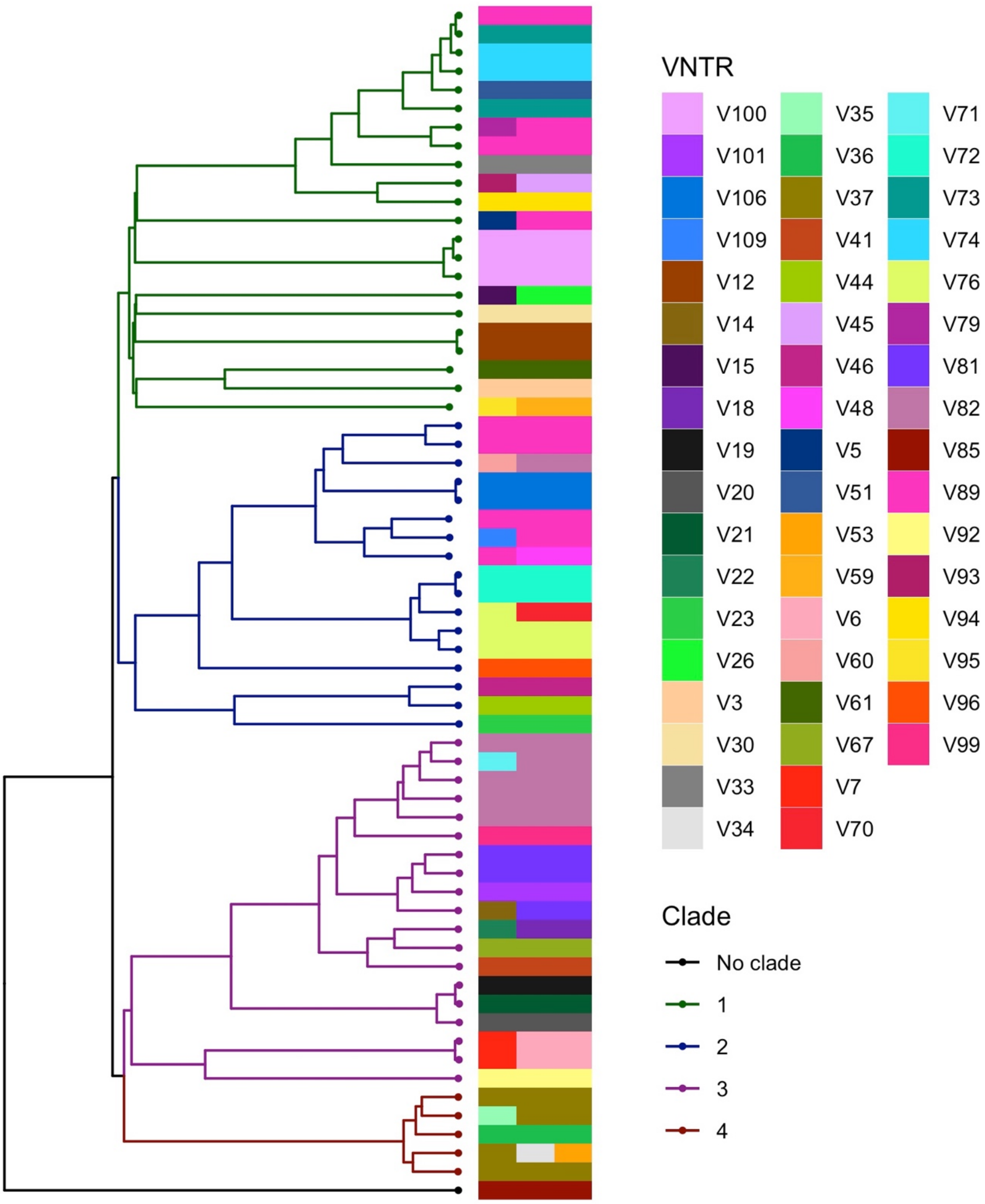

Figure S6: Visual comparison between the *M. bovis* phylogenetic MCC tree and the MIRU-VNTR types. Fifteen isolates were associated with two MIRU-VNTR types, and one with three, because multiple samples from the same animal (up to three) were submitted for molecular typing.

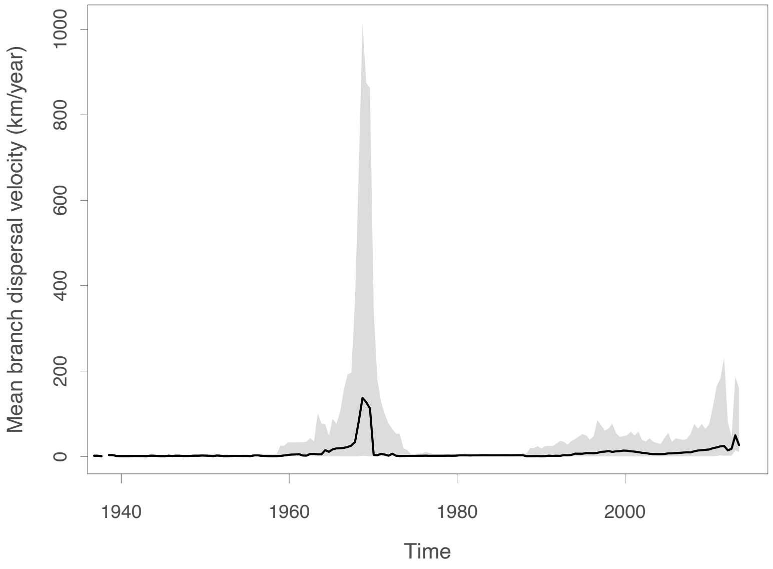

Figure S7: Mean (and 95th HPD) branch velocity evolution in time estimated by BEAST. The velocity jump of 2008 can be associated with the rapid expansion of *M. bovis* in the outbreak area. After 2010 it is possible to observe a slow but steady increase in velocity.

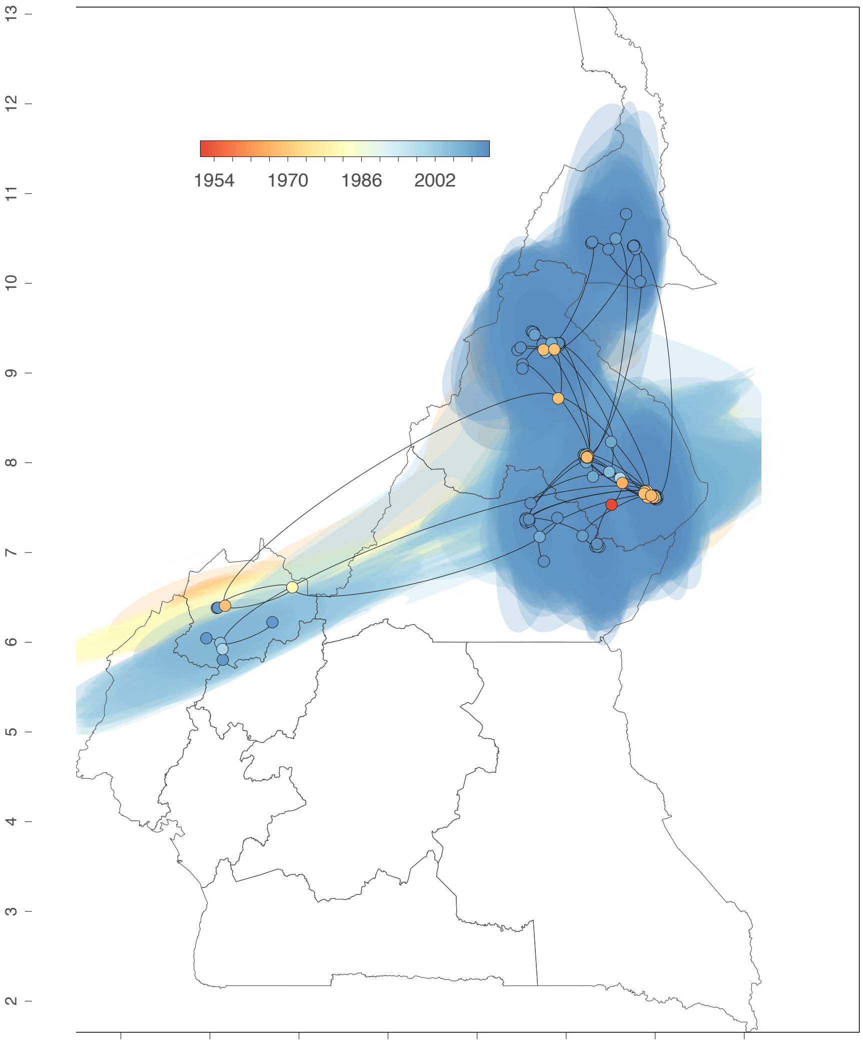

Figure S8: Mean (and 95th HPD) of the internal nodes' locations according to the maximum clade credibility trees estimated by BEAST. Nodes and shades are coloured according to their point in time estimates, while shades size accounts for the 95th HPD of the internal nodes' location.

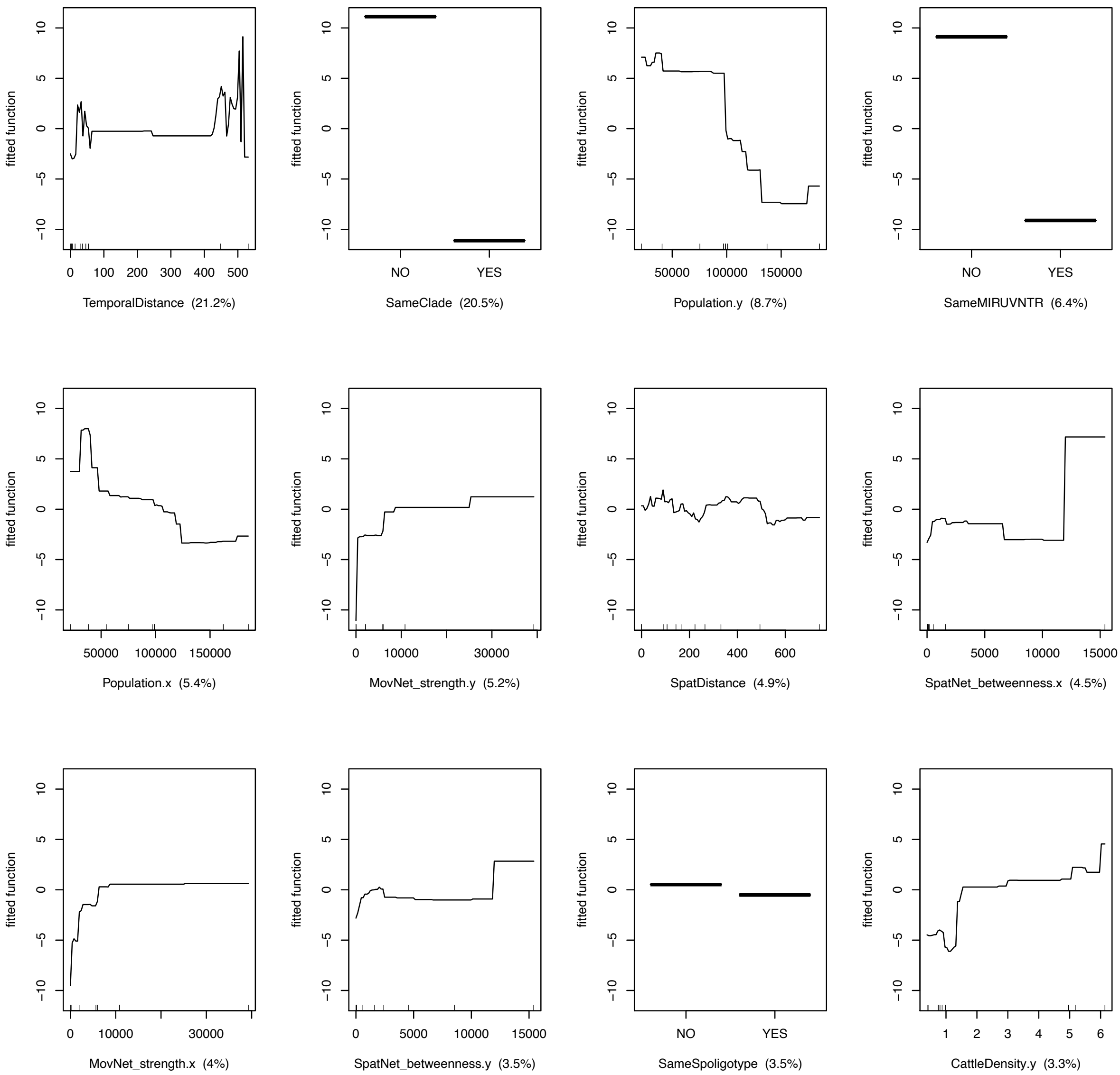

Figure S9: The partial dependency plots showing the effect on the SNP distance of the twelve most important variables, identified by the boosted regression trees model (in parenthesis the variables' influence on the model).
